# DANSE: a pipeline for dynamic modelling of time-series multi-omics data

**DOI:** 10.1186/s12859-025-06354-3

**Published:** 2025-12-30

**Authors:** Lucas F. Jansen Klomp, Xinqi Yan, Rebecca R. Snabel, Gert Jan C. Veenstra, Hil G. E. Meijer, Janine N. Post

**Affiliations:** 1 Department of Applied Mathematics, Faculty of Electrical Engineering, Computer Science, Mathematics, University of Twente, Drienerlolaan, 7522NB Enschede, The Netherlands; 2 Developmental BioEngineering, Faculty of Science and Technology, University of Twente, Drienerlolaan, 7522NB Enschede, The Netherlands; 3 Department of Molecular Developmental Biology, Radboud Institute for Molecular Life Sciences, Faculty of Science, Radboud University, Houtlaan, 6525XZ Nijmegen, The Netherlands

**Keywords:** Multi-omics, Time series, ODE modelling, Data-driven, Mechanistic modelling, Gene regulatory network

## Abstract

**Background:**

Understanding time-dependent intracellular processes, such as cell differentiation, is key to developing new therapies for a wide range of diseases. Models that connect transcription factor activity to dynamic expression patterns are rare, despite the increased availability of time-series data.

**Results:**

To identify key regulators of time-dependent biological processes, we present the pipeline DANSE: Dynamics inference Algorithm on Networks Specified by Enhancers. Starting from multi-omics data, our pipeline constructs a data-driven mechanistic transcription factor (TF) network and subsequently defines a dynamic model based on this TF network. The combination of a TF network and a mechanistic model allows for the identification of a small set of key transcription factors predicted to drive the modelled biological process. We showcase the result of our pipeline by applying DANSE to two different datasets that describe iPSC differentiation.

**Conclusions:**

Models constructed using DANSE suggest testable hypotheses for the perturbation of gene expression, for example, knockdown or overexpression, that influence cell fate. In this way, DANSE is a powerful tool for generating novel hypotheses in a data-driven manner that take into account the dynamic nature of multi-omics time series data.

## Background

Time-dependent intracellular processes, such as cell differentiation, are governed by complex interactions between signalling pathways and transcription factor (TF) networks. In recent years, time-series multi-omics data have provided a way to elucidate these temporal processes in the cell. Many methods of analysing these data have been proposed, including differential expression analysis, cell annotation and clustering, as well as forecasting new time points from existing data [1, 2]. However, their outputs do not directly provide mechanistic insight into which key genes control temporal changes in expression levels. To gain such mechanistic insights, dynamic models based on gene regulatory networks (GRNs) have been proposed [3–5]. The construction of such dynamic models first requires the inference of a suitable gene regulatory network. This task requires specialised inference methods that focus specifically on mechanistic connections between genes.

In literature, many approaches have been proposed to infer gene regulatory networks from omics data [6–8]. Originally, these approaches focused on inferring networks based on bulk RNA-seq data [9, 10]. Recently, the focus has shifted to using multi-omics data [11–13] for GRN inference. Data collected at intracellular levels other than the mRNA level are often used to identify relationships between transcription factors and cis-regulatory elements, such as enhancer or promoter regions [6]. The benefit of these approaches is that there is more evidence for each inferred connection, leading to a clearer biological interpretation of the inferred networks. Common methods for inferring GRNs from multi-omics data include SCENIC+, which focuses on retrieving sets of a transcription factor (TF) and their targets from single-cell multi-omics data [14]. The method GraNIE infers both TF-peak and peak-gene links from RNA-seq and ATAC-seq data [12]. In this work, we consider the network inference method ANANSE [11] that uses a prediction of TF-enhancer binding to infer a GRN. The method ANANSE is particularly interesting for investigating time-dependent data, since the method is built to find key transcriptions involved in trans-differentiation. As a validated method to determine key genes distinguishing different cell fates, ANANSE is a promising tool for the construction of cell-type specific networks throughout cell differentiation.

Gene regulatory networks are often used as a basis for dynamic models, which provide additional mechanistic insight. In some inference methods, a gene regulatory network is inferred jointly with its dynamics [15–18]. However, these methods often focus on the inference of the gene regulatory network, rather than on dynamics. Other works describe how a predefined GRN is converted to an ordinary differential equations (ODE) model that mimics experimental data by fitting kinetic parameters [3, 4]. Some ODE models have been used to describe and identify intracellular mechanisms in cell fate decisions and signalling pathways [19, 20]. Though ODE modelling forms a natural paradigm for exploring gene expression over time, current methods are often focused on modelling known biological processes, reducing the ability to find key novel markers.

Here, we present DANSE: **D**ynamics inference **A**lgorithm on **N**etworks **S**pecified by **E**nhancers. In this pipeline, we use TF networks inferred using ANANSE to select key TFs from data and to infer a baseline gene regulatory network that indicates probable connections between the selected transcription factors. Subsequently, we infer a dynamic model based on this gene regulatory network, finding a minimal network that mimics the gene expression dynamics of modelled TFs. Through this process, we refine the inferred gene regulatory network and construct a mechanistic model of the temporal process described in the experimental data. This model provides direct mechanistic insights into possible drivers of the temporal process through varying parameters in the model. While many current popular analysis methods result in large sets of important TFs, the analysis of our dynamic models results in the identification of prioritised short lists of TFs based on temporal dynamics, which are interesting for conducting knockdown or overexpression experiments in a wet-lab setting. We tested the performance of our pipeline with two multi-omics datasets: one describes pluripotent stem cells differentiating toward chondrocytes, while another dataset describes differentiation toward epicardioids [21–23].

## Methods

**Fig. 1:**
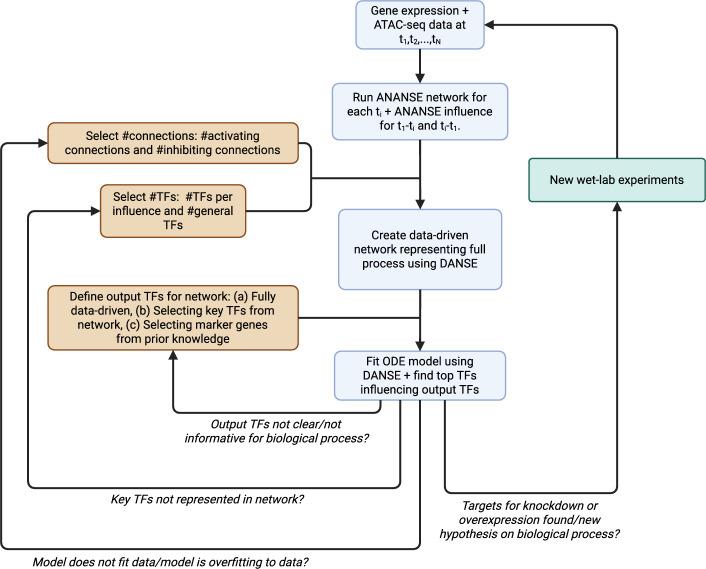
Flowchart for using our proposed pipeline, DANSE. The required input data is time series multi-omics data of an intracellular biological process. Three user-defined inputs are needed: the number of transcription factors and interactions desired for the computational model and the choice for output TFs used to rank TFs in the network. The selected TFs are divided into two groups: TFs that are generally important as they are densely connected in all constructed core TF networks (#general TFs), and TFs that are identified from comparisons between the first time point and each subsequent time point (#TFs per influence). The DANSE pipeline then constructs a computational model based on the input. Subsequently, if the model needs adjustment, the choice for the number of TFs or the number of interactions can be adjusted. If not, the output of the model can be used to suggest knockdown or overexpression experiments, along with predicting key drivers underlying the considered process. If desired, new computational models can be generated based on the results of these experiments

The DANSE pipeline requires several inputs. First, multi-omics data at various time points are used to build the data-driven model. Second, the desired number of activating connections, inhibiting connections and transcription factors should be indicated for the generated model. Finally, a choice for the output TFs used to determine important transcription factors in the network is required. Based on these inputs, DANSE provides the functionality to build a computational model for the biological process described by the data, along with the capability to suggest drivers underlying these processes. Below, we describe and discuss all the choices made in this process of constructing a data-driven model based on multi-omics time series data. For a flowchart describing the workflow when using DANSE, we refer to Fig. 1.

**Fig. 2:**
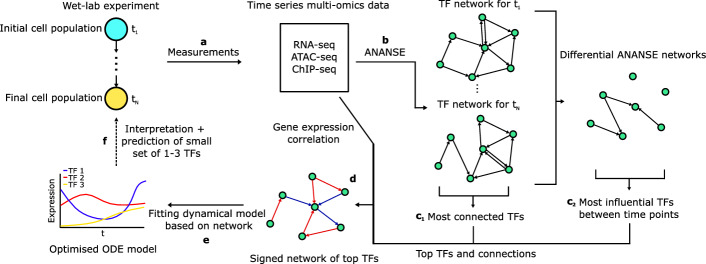
Overview of the DANSE pipeline for making dynamic models based on time-series multi-omics data. Multi-omics data in the form of gene expression data, ATAC-seq data and optionally ChIP-seq data is used to infer a GRN that is suitable for dynamic modelling using the GRN inference tool ANANSE. Subsequently, an ordinary differential equations model is defined based on this network and parameters in this model are optimised, leading to a minimal model that describes the dynamics found in the original experiment (a schematic simulation of TF expression is shown here). Insights and mechanisms found in this computational model can be used to inform decisions on new experiments, leading to a modelling cycle

### Network construction

The first step of our pipeline is the construction of a suitable gene regulatory network on which to base the dynamic model. Such a suitable network needs to include connections that are mechanistically realistic. Moreover, such a network should consist of somewhere between 10–50 nodes, as networks with too many nodes may be hard to interpret, while too few nodes may lead to missing essential regulators. Finally, the constructed network should include both activating and inhibiting connections. Including inhibiting connections in the network allows for the construction of more types of feedback loops in the network, which are key to describing specific behaviour using dynamic models (for example, bistability or oscillations).

To construct such a network, we first construct core transcription factor networks for each available time point using ANANSE, using the RNA-seq data and ATAC-seq and/or ChIP-seq data collected at those time points as input (step b in Fig. 2). For each TF network constructed for the individual time points, the interaction score $A_{ij}$ between TFs *i* and *j* is the connection strength defined using ANANSE:

$$\begin{aligned} A_{ij} = \frac{1}{4}(S(E_i) + S(E_j) + S(A_i) + S(B_{ij})). \end{aligned}$$

Here, $E_i$ $(E_j)$ denotes the average expression of TF *i* (*j*) at the considered time point, $A_i$ denotes genome-wide activity of TF *i* and $B_{ij}$ denotes inferred binding probability of TF *i* to enhancer regions close to the gene associated with target TF *j* [11]. The function *S* scales each value to the interval [0, 1]. To infer the genome-wide TF activity, motifs are ranked based on their predictive value for the ATAC-seq/ChIP-seq data using ridge regression. To determine the binding probability $B_{ij}$, an inferred probability of the binding of TF *i* to an enhancer region close to the target gene *j* is inferred based on the available ATAC-seq data and binding motifs of TF *i*. Subsequently, a weighted sum per enhancer region is used to calculate $B_{ij}$. In this weighted sum, enhancer regions that are further away from the promoter region get a lower weight. The result is a fully connected network representative of one time point in which interactions between both TFs and target genes are included.

We then use the influence score implemented in ANANSE to identify TFs that change significantly in importance between time points [11]. This influence score is based on changes between time points in network connectivity and expression levels. Specifically, a differential network is first constructed that includes only interactions for which the interaction score increased between $A_{ij}$ for a considered time point and $A_{ij}$ for another time point. For transcription factors, this differential network is used to assess changes in connectivity: if genes close to a TF, defined by the length of the shortest path in edges in the differential network, have a high change in expression the influence score increases [11]. Hence, it is a measure that identifies TFs that are important in one time point compared to another. By including TFs with a high influence score in our model, we aim to identify TFs that have an important role in one or more specific stages during differentiation.

To select TFs for our network, we compare the network of each individual time point with the network for the first time point in the differentiation experiment. In this way, we aim to identify TFs that best explain the differences between these cell states. We computed the ANANSE influence score between each time point and the first time point both forward and backward in time. In this way, we identified TFs that are more important at each later time point compared to the first time point and TFs that are more important at the first time point compared to each later time point. For each comparison, we include the top genes ranked by influence score in our network (step c$_2$ in Fig. 2, Figure A1). This yields a set of genes per time point after the first time point. In order to include TFs that are important throughout the entire differentiation process, we also included in our network topology the top TFs ranked by the average number of network connections over all time points (step c$_2$ in Fig. 2). We note that while ANANSE’s influence score explicitly considers only TFs, selecting nodes by degree does not. However, as TFs are the only nodes with outgoing connections, it is unlikely that genes other than transcription factors are selected using our methodology. Finally, we remove all duplicate genes from our set of selected TFs, ensuring each TF only appears once in the gene regulatory network.

Connections between the TFs are selected based on the static networks constructed for each of the considered time points (step e in Fig. 2). To accommodate potential context-dependent effects based on the composition of regulatory elements, we designed our model such that TFs could have variable effects on target genes. Therefore, we first sign each possible connection, defining them as activating or inhibiting based on the correlation between gene expression of the source and target TFs. We then select the top activating and inhibiting connections as defined by the maximum of the interaction scores over all time points for which a network is made.

We use a different interaction score for the inhibiting connections (as defined by a negative correlation in gene expression), where for the expression of the target TF *j*, a low expression level is preferred, so:

$$\begin{aligned} A_{ij} = \frac{1}{4}(S(E_i) + (1-S(E_j)) + S(A_i) + S(B_{ij})). \end{aligned}$$

We note that binding of TFs to enhancer regions is generally associated with upregulation of target genes. However, silencers, the inhibition-associated counterparts to enhancers, are not as well documented. Therefore, we have chosen the above formulation to be able to identify potential inhibiting connections which are key for modelling, but we emphasise that these connection strengths are not mechanistically accurate.

Based on these interaction scores inferred at different time points, we select the time point at which the genome wide TF activity and binding score is maximal, and we select the top $n_a$ activating connections and the top $n_i$ inhibiting connections based on the static networks constructed for these time points. Subsequently, we only keep those nodes in the network that have at least one incoming activating connection, removing all others, and repeat until no such nodes remain in the network. Hence, in our pipeline, the potential nodes in the network are selected first, after which the connections are iteratively added to the network. Because we remove nodes that get no activating input after selecting connections, the number of nodes in the final TF network depends on the number of activating connections chosen. The selection of the number of connections should be based on the resulting dynamical model. We comment on this in more detail when presenting the results of the application of DANSE.

### Dynamic modelling

Based on the constructed TF network, the aim of the DANSE pipeline is to describe time-series data and explore mechanisms underlying the data. To do so, we formulate an ordinary differential equations model that describes the change in expression of each transcription factor $x_i$ (Fig. 2e). Each TF decays naturally over time and its activation or inhibition by other TFs is modelled by a Hill function. The ODE system is given by:

1 $$\begin{aligned} \tau \dot{x_i} =&-x_i + \frac{\sum _{j\in V_{\text {act}}}W_{ji}x_j^n}{S^n + \sum _{j\in V_{\text {act}}}W_{ji}x_j^n + \sum _{j\in V_{\text {inh}}}W_{ji}x_j^n}. \end{aligned}$$

In this system, $x_i$ denotes the expression of TF i, whereas $\dot{x}_i$ denotes the time derivative of $x_i$. The weight matrix $W_{ji}$ denotes how strongly TF *j* influences *i*. As such, the matrix *W* describes the connection strength for the entire network. If there is no connection between TF *i* and *j*, $W_{ij} = W_{ji} = 0$. The sets $V_{\text {act}}$ and $V_{\text {inh}}$ denote the sets of TFs that activate and inhibit TF *i* respectively. As the activating influences appear both in the numerator and denominator, the Hill function will increase if these influences become stronger, while the Hill function will decrease if the inhibiting influences become more active. The parameter *S* denotes the half occupation value, and *n* is the Hill coefficient, determining the steepness of the Hill function. We set $S=1$ and $n = 3$, leading to a reasonably steep sigmoid shape. Such a steep sigmoid leads to easier representation of bistability within the system. The parameter $\tau$ describes the time scale of the full system.

Models based on Hill functions are widely used in literature to model gene regulatory networks [24, 25]. The specific form of the Hill function used in our pipeline is also described in literature [19] and has several desirable properties. First, because the Hill function is bounded between 0 and 1, the activity of a modelled TF will stay between 0 and 1 if its initial conditions are between 0 and 1. This is seen through the fact that $\dot{x}_i \ge 0$ if $x_i = 0$ and $\dot{x}_i \le 0$ if $x_i=1$, provided that $x_j \ge 0$ for all other TFs *j* and $W_{kj} \ge 0$ for all *k*, *j*. This allows for modelling normalised and scaled RNA-seq data. Second, this formulation allows for large effects of a single activating or inhibiting input in the absence of other influences. Because of this, our formulation is less sensitive to differences in the amount of incoming connections for each node. We use a single connection strength for each connection in the network, and do not adjust the connection strength according to changing chromatin accessibility over the full temporal process.

### Parameter optimisation

We optimise the weight matrix *W* in the ODE model to fit time-series expression data of the modelled TFs (Fig. 2e). Given data $D_{i}(t)$ denoting the expression of TF *i* at time point *t* and a solution $\phi (t,W) \in \mathbb {R}^{N}$ for a given *W* to the system of *N* ODEs given by Equation 1 with $\phi _i(0,W) = D_i(0)$, we optimise *W* by numerically minimising a loss function given by:

2 $$\begin{aligned} L(W) = \sum _{t_k}\sum _{i = 1}^{N} (\phi _i(t_k,W) - D_{i}(t_k))^2 + C\sum _{i=1}^N\sum _{j=1}^N|W_{ij}|. \end{aligned}$$

Here, $\phi _i(t,W)$ denotes the *i*th element of $\phi (t,W)$ and therefore describes the expression of TF *i* at time *t*. The first term in this loss function denotes the squared error between the expression level data and the simulation of the ODE system (1). For each connection between TFs *i* and *j* that is not part of the full inferred gene regulatory network, we fix $W_{ij} = 0$; these elements of the weight matrix are not optimised. We note that *W* is static throughout the simulation of the model.

The second term in the loss function is an L1 regularisation term. This term penalises any nonzero elements in the weight matrix. The penalty is multiplied by a scalar $C \in \mathbb {R}$. By setting a low value for *C*, $C = 0.005$, we ensure that connections in the gene regulatory network that do not contribute significantly to fitting experimental data are removed. On the other hand, taking a low value for *C* ensures that a good fit of the data is prioritised. In turn, the L1 regularisation term will only remove connections that do not add anything to the fit of the model, or connections that have the same function as other connections in the network. In this way, our optimisation procedure looks for a minimal network structure that simulates the experimental data with an added restriction that any connection included in the model must be feasible based on the given experimental data, based on the original constructed TF network.

We optimise the loss function using the gradient-based optimiser ADAM with learning rate 0.1 [26]. We note that this is a high learning rate, but we have found that lower learning rates do not yield better fitting results or more stable training behaviour in our test cases.

A choice made during the preprocessing of expression level data is how this data should be scaled to give as input for the parameter fitting procedure. DANSE takes normalised expression levels as input, and scales the data so that expression levels for all genes fall within the interval [0, 1]. That is, we compute the scaled expression level of gene *i* at time *t*, denoted by $X_{i,t}$, from the normalised expression level $\hat{X}_{i,t}$ by:

$$\begin{aligned} X_{i,t} = \frac{4}{5(M_i-m_i)}(\hat{X}_{i,t} -m_i)+0.1, \end{aligned}$$

where $M_i$ and $m_i$ are the maximum and minimum expression levels for gene *i*, respectively. The normalisation procedure for the data depends on the data type used as input. For examples, we refer to Sect. 2.5 for the datasets used in this paper. This scaling procedure scales the expression of all genes between 0.1 and 0.9, where the maximum expression level of a gene is assigned a scaled expression of 0.9, and the minimum expression level is assigned a scaled expression of 0.1. From a modelling perspective, this ensures that there is ample variation in gene expression for each modelled gene, and therefore the model cannot “ignore” particular genes in the network. As only highly active TFs and TFs with high changes in activity are included in the model, stable lowly expressed genes are not highlighted in an undesirable way using this procedure. From a biological perspective, genes with low expression levels may still have a significant influence on the rest of the system, hence why alternative methods such as scaling over all genes are not viable options.

A choice made prior to running the DANSE pipeline, the choice of the number of connections in the model, can be based on the results from fitting the model. Specifically, the aim is to select the least number of connections necessary to obtain a reasonable fit of the data, hence aiming to avoid overfitting of the data (here defined as parsimony) or having too many options for recreating the data using the selected network. A selection of too many connections may in some cases lead to biologically unfeasible behaviour such as oscillations. To determine this threshold, we recommend to optimise the model multiple times for different choices of the number of activating connections $n_a$ and inhibiting connections $n_i$. In this way, a grid search is performed for the choice of the number of connections in the network. We consider the average value of the loss function for all nodes in the network to be an indicator of the fitting performance:

$$\begin{aligned} L_{\text {av}}(W) = L(W)^2/N \end{aligned}$$

where *N* denotes the number of nodes in the network for that particular optimization. If this metric drops significantly when adding new connections to the model, this is an indication that the fit has performed well. We describe other criteria for selecting these parameters when discussing the results for our case studies.

### Identification of key transcription factors

As a method of obtaining novel insights based on the constructed dynamic model, we focus on the discovery of key transcription factors (Fig. 2e). The metric we use to determine the importance of a TF relies on how much upregulation or downregulation of this TF influences the expression of selected key TFs: transcription factors associated with an on-target cell fate. Upregulation and downregulation of TFs is modelled by modifying the right-hand side of the ODE model for a TF *i* that is upregulated or knocked down:

3 $$\begin{aligned} \tau \dot{x_i} =&-x_i + \frac{p_{\text {act}}+\sum _{j\in V_{\text {act}}}W_{ji}x_j^n}{S^n + p_{\text {act}} + p_{\text {inh}}+ \sum _{j\in V_{\text {act}}}W_{ji}x_j^n + \sum _{j\in V_{\text {inh}}}W_{ji}x_j^n}. \end{aligned}$$

Here, $p_{\text {act}}$ and $p_{\text {inh}}$ are the controlled variables used to indicate how much TF *i* is upregulated or knocked down. When $p_{\text {act}}=p_{\text {inh}}=0$, there is no change compared to the original model. When $p_{\text {act}} > 0, p_{\text {inh}}=0$, transcription factor *i* is upregulated. On the other hand, when $p_{\text {inh}} > 0, p_{\text {act}} = 0$, transcription factor *i* is downregulated. In this controlled case we do not consider the case where $p_{\text {act}}$ and $p_{\text {inh}}$ are both non-zero. Specifically, we modify one parameter *p* so that

$$\begin{aligned} {\left\{ \begin{array}{ll} p_{\text {act}} = p, p_{\text {inh}} = 0 \,\,\,\, \text {if }p \ge 0,\\ p_{\text {inh}} = -p, p_{\text {act}} = 0 \,\,\,\, \text {if }p < 0. \end{array}\right. } \end{aligned}$$

We then define a set of transcription factors $T = \{i_1, i_2,...,i_n\}$ of transcription factors $i_k$ that are known or inferred to be markers for the modelled biological process associated with the desired cell fate, selected from the TFs present in the inferred TF network, from data or from prior knowledge. If we perturb TF *j* setting $p = p_j$, we let $\phi _i(t,p_j)$ denote the simulated expression of TF *i* at time *t*, where TF *j* is simulated using Equation (3), and all other TFs are simulated following Equation (1). For each TF in the network, we then find:

$$\begin{aligned} z=\max _{p} \sum _{i\in T} \phi _i(t_N,p_j) - \phi _i(t_N,0). \end{aligned}$$

Here, $t_N$ denotes the final time point of the time series data used to fit the original model without perturbation. This maximum *z* is a metric for how much the regulated transcription factor influences the set of transcription factors *T*. In this metric, we focus only on upregulation of the target genes in *T*, since the selected set of known transcription factors are assumed to be desirable for a specific cell fate. In the practical applications presented in this paper, we consider $p\in \{-3,-2\frac{19}{20},-2\frac{18}{20},...,2\frac{19}{20},3\}$ to estimate *z*. By calculating our metric for each node in the network, we can obtain a list of the main transcription factors that influence specific markers according to the computational model constructed using DANSE. Our method will present a prioritised set of TFs of interest, eliminating the need to sift through large sets of identified transcription factors. We highlight here that the novelty of our selection method is that it takes the temporal dynamics into account, which differs from methods using only one time point. Moreover, since we have the underlying computational model, we can subsequently identify an underlying mechanism explaining how this particular TF may upregulate the set of selected target transcription factors. In some cases, these effects can be indirect and are only clearly visible through the introduced dynamics. In this way, the DANSE pipeline provides a novel way of generating hypotheses based on multi-omics time series data. We highlight here that the difference between classical gene expression analysis and our method is that our method of finding key TFs is explicitly based on the constructed mechanistic dynamic model.

We provide three options to choose the set of key transcription factors *T*. First, TFs can be selected in a data-driven way. Here, the TFs that return most often in influence scores calculated using ANANSE going forward in time are added to the network as output nodes. If these nodes are not already in the TF network, the top two incoming activating connections and the top two incoming inhibiting connections are added based on the ANANSE interaction score. Second, TFs can be selected directly from the final TF network by hand. This is a good option if TFs are included in the network which have a well-known function within the modelled biological process. Finally, TFs can be selected from prior knowledge. Similar to the data-driven option, the top two incoming activating and the top two incoming inhibiting connections to these TFs are included in the network. This option is most relevant if one wants to investigate the regulation of particular marker genes known from other work.

Since our model optimisation procedure yields slightly different optimised models in multiple runs, we suggest running the optimisation multiple times and recording the importance measure *z* for each TF in the network for each individual fit. By measuring statistics of the importance of the TFs on target genes of interest over different runs, it is possible to quantify the robustness of our method. We showcase this procedure in the two case studies in the Results section.

### Data

In this section, we describe in detail the preprocessing steps used to get to the input data used for DANSE for the two computational models presented in this work for chondrogenesis and cardiogenesis.

#### Preprocessing for chondrogenesis data

In the chondrogenesis experiment conducted by Kawata et al. [21], microarray and bulk ATAC-seq data were collected for early time points during differentiation from hiPSCs towards chondrocytes. Data was obtained from the NCBI Gene Expression Omnibus public repository (https://www.ncbi.nlm.nih.gov/geo/, accession numbers GEO: GSE96036, GSE109172 and GSE132532). The raw microarray data were log2 transformed, and subsequently normalised using limma (normalizeBetweenArrays) [27], using the settings needed for Agilent microarray chips. In some cases, multiple probes were associated with one gene, in which case we have used the first found probe. For each time point, three samples were available and we averaged them. These averaged expression levels were used as input for our network construction and parameter optimisation. For the differential expression data needed to compute the ANANSE influence score, the log2 fold change in expression between time points was computed. Given the available data, we used a single t-test for determining the significance without further correction.

The deposited ATAC-seq data contains peak locations derived from the raw data. For each time point, we converted the data to a count matrix, a format supported for network construction using ANANSE [11]. The resulting count matrices were used as input for network construction using ANANSE.

#### Preprocessing for epicardioid differentiation data

In the differentiation experiment towards epicardioid conducted by [23] , single-cell data were collected during a differentiation experiment from hiPSCs towards epicardioid on days 2, 3, 4, 5, 7, 10, and 15. Data were obtained from the NCBI Gene Expression Omnibus public repository (https://www.ncbi.nlm.nih.gov/geo/, accession number GEO: GSE196516). For quality control in single-cell RNA-seq data, the R package Seurat [28] was used to exclude cells with unique feature counts over 7500 or less than 200, as well as cells with a percentage of mitochondria genes greater than 25. We then employed the “LogNormalize” method with a scaling factor of 10000. To use expression data for ODE modelling, we then converted the expression level of each day into bulk data by taking the average expression of genes across all cells. For differential expression analysis on scRNA-seq data, we compared other days with day 2 using a Wilcoxon test and took the adjusted p-value based on a Bonferroni correction and fold change as input for ANANSE. For single-cell ATAC-seq data, we used the Signac [29] package for quality control, we first annotated peaks using Ensembl database[30]. We then varied the number of peaks to exclude low-quality cells with a transcriptional start site (TSS) enrichment score less than 1, or an approximate ratio of mononucleosomal to nucleosome-free fragments greater than 1.2, or ratio reads in genomic blacklist regions greater than 0.006. Table 1 represents the number of cells before and after QC, as well as parameter choices. Through this preprocessing procedure, we obtained the required inputs for running ANANSE to make the TF networks used in DANSE.

**Table 1:** Metric choices during ATAC-seq data preprocessing

Days	# cells before QC	# cells after QC	nCount_peaks
D2	7051	6986	9k–75k
D3	6755	6597	9k–75k
D4	5019	4689	9k–75k
D5	1651	1519	9k–200k
D7	669	613	9k–200k
D10	3116	2434	9k–100k
D15	5068	1946	9k–100k

## Results

We illustrate the use of the DANSE pipeline by applying it to two publicly available datasets. Specifically, we ask whether it is possible to obtain novel biological insights into pluripotent stem cell differentiation using our pipeline. We consider a dataset describing chondrogenesis and a dataset describing epicardioid differentiation.

### Chondrogenic differentiation

To explore chondrogenesis, we have applied DANSE to a dataset generated from a chondrogenesis experiment conducted by Kawata et al. [21]. From this experiment, microarray and bulk ATAC-seq data is available for days 0, 2, 3, 4, 5 and 9 during differentiation. The differentiation relied on a combination of two compounds, a WNT activator and retinoic acid, to differentiate iPSCs towards chondrocytes. The raw data has been preprocessed before being used as input for DANSE. For details on this preprocessing procedure we refer to Sect. 2.5.

To construct a model using DANSE for this dataset, we selected the top 5 genes per comparison between day zero and a later time point, and selected the top 12 generally important transcription factors (Figure D9). We chose to include the top 70 activating connections and the top 40 inhibiting connections in the network. This was based on rerunning the pipeline for various choices for the number of activating and inhibiting connections (Supplementary Material A, Figure A2). At the threshold of 70 connections, the fit of the model improved noticeably compared to adding fewer activating connections as shown in Figure A2.

**Fig. 3:**
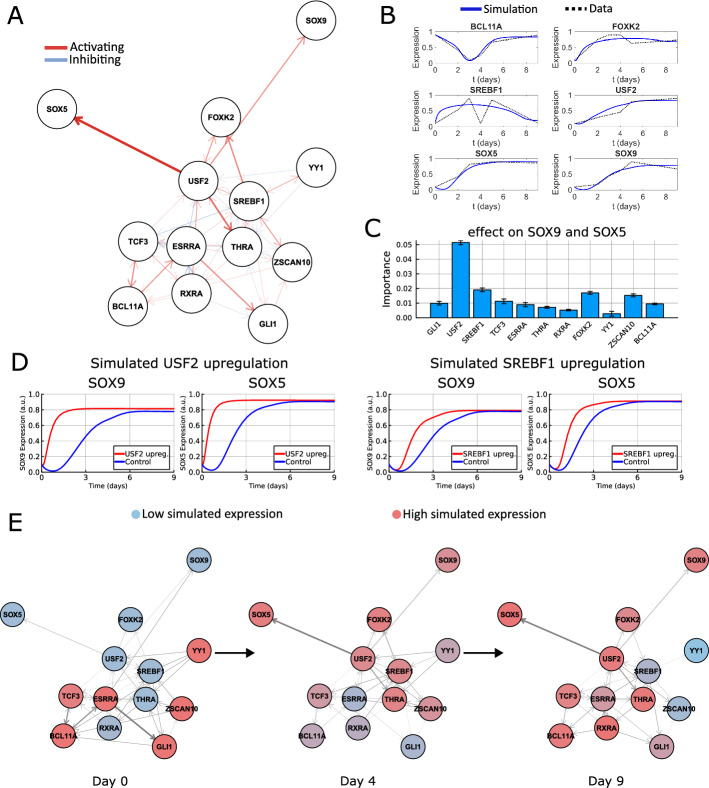
Computational model constructed using DANSE based on the data from Kawata et al. [21] **A**. Constructed TF network based on the multi-omics data describing the full differentiation process. Here, activating connections are indicated in red and inhibiting connections are indicated in blue. The thickness of each connection indicates the strength of that connection in the fitted dynamic model. The position of the nodes is not related to the model **B**. Simulations of the fitted dynamic model, showing a qualitative match between model simulations and gene expression level data. **C**. Calculated importance measures for all TFs showing which TFs in the network have the most effect on SOX9 and SOX5 expression when knocked down or overexpressed. (caption continues on next page) **D**. Simulated upregulation experiments based on the identified driver transcription factors. The effects of simulated upregulation of USF2 and SREBF1 on SOX9 and SOX5 expression throughout a simulation of the model are shown. **E**. Visualisation of expression levels over time in the TF network. The colors of the nodes indicate simulated expression levels of the TFs at the depicted time points. The thickness of the edges depicts how much signal is going through each connection, defined as the activity of the source node multiplied by the fitted connection strength

By running the DANSE pipeline, we obtained a dynamical model for the chondrogenesis process that mimics the gene expression profiles of most genes in the data (Fig. 3B). The fit for some genes, such as SREBF1, is not perfect. In the case of SREBF1, this can be explained from the fact that YY1 is solely activated by SREBF1. Looking at the data, some genes, including SREBF1, have a very low variance in their expression profile, which is amplified by the scaling procedure used. The constructed data-driven TF network contained various important known TFs (Fig. 3A). The transcription factors RXRA and TCF3 are directly related to retinoic acid signalling and WNT signalling, the two stimuli applied in the protocol described by [21]. Other genes in the network, such as ESRRA, are known to play a role in chondrocytes [31, 32]. The constructed model did not contain clear marker genes for chondrogenesis, and for this reason we have manually added SOX9 and SOX5 to the model as output genes. Both SOX9 and SOX5 are well-established chondrocyte markers markers, and are used to assess on-target differentiation in the dataset of Kawata et al. [21, 33–35]. This makes understanding the TFs regulating these targets particularly interesting. The model also included some genes that have less well-defined roles within chondrogenesis, such as SREBF1 and BCL11A.

To obtain potential targets influencing the chondrogenesis process, we asked which other factors in the constructed model are promising targets for modulating the expression of SOX9 and SOX5. Our data-driven model suggested that many TFs in the network have some effect on the expression of SOX9 and SOX5. However, USF2, SREBF1, and FOXK2 are the most promising targets following our importance metric (Fig. 3C). Our model suggests all three of these TFs should be upregulated to increase the expression levels of SOX9 and SOX5 (Figure B7). In literature, one of these genes has a known function in cartilage differentiation: the gene USF2 is known to play a role in cartilage differentiation, though its role is described as promoting osteogenic differentiation and inhibiting cartilage differentiation [36, 37]. Less is known about the function of SREBF1 and FOXK2 in chondrogenesis, making these potential new targets for knockdown or overexpression experiments.

Our result persists locally if small changes are made in the number of selected activating or inhibiting connections. Being the direct regulator of SOX9 and SOX5 in the model, USF2 is consistently found to be the TF for which knockdown or upregulation leads to the biggest change in activity of SOX9 and SOX5 for different network configurations (Figure A3). The TFs FOXK2 and SREBF1 are often found to be the second most important TFs in the network for influencing SOX9 and SOX5 expression, though other TFs are also found to be important for specific network configurations. We then looked at the effect of the scaling procedure on the importance metric for genes with low variance in gene expression (Supplementary Material C, Figure C8). This experiment showed that the driver TFs change if the input expression level data changes. However, USF2, SREBF1 and FOXK2 are still often recovered as top choices for driver TFs.

Novel biological hypotheses can be formulated based on the identification of these important transcription factors in our computational model. Taking SREBF1 as an example, the fitted TF network suggested that GLI3 is affected indirectly by SREBF1 through the activation of FOXK2 and USF2, the other TFs found to be important. As such, the application of DANSE resulted in testable hypotheses on the workings of intracellular dynamics, which can be validated in wet-lab experiments through knock-down and overexpression experiments. Specifically, the model suggested the upregulation of USF2, SREBF1 and FOXK2 to be beneficial to increasing the expression of SOX5 and SOX9. In simulated upregulation experiments, the effects on the final expression of SOX9 and SOX5 were marginal, but both genes are expressed much earlier than in simulations without upregulation of USF2 and SREBF1 (Fig. 3D). While the TFs included in the computational model were based on the influence score computed using ANANSE, the order in which TFs are ranked using DANSE is different from when ANANSE is used directly. This shows how the construction of the computational model leads to novel insights into the gene regulatory network structure. In general, the focus of many existing methods is to find genes that are differentially expressed or have different activity across cell types, whereas our method aims to find the regulators of these differentially expressed genes or differentially active transcription factors.

### Epicardioid differentiation

To illustrate the use of our pipeline for exploring single-cell data, we applied DANSE to a single-cell multi-omics dataset which describes iPSC differentiation to epicardioid cells [23]. The data describes the formation of self-organizing human pluripotent stem cell-derived epicardioids that display patterning of the epicardium and myocardium. scRNA-seq and scATAC-seq data were collected on day 2, 3, 4, 5, 7, 10 and 15 during the differentiation experiment.

To construct the dynamic model describing the epicardioid differentiation process, we selected the top 5 genes per comparison between day two and a later time point, and selected the top 12 generally important transcription factors (Figure D11). We then selected the number of activating and inhibiting connections for the model in order to obtain a good fit of the model to the data. From the optimization results in Figure A4, the model performed well when 130 activating connections and 20 inhibiting connections were included. If fewer activating connections are included, the fit performs considerably worse, so 130 activating connections provided a choice for a minimal model that matches the experimental data.

**Fig. 4:**
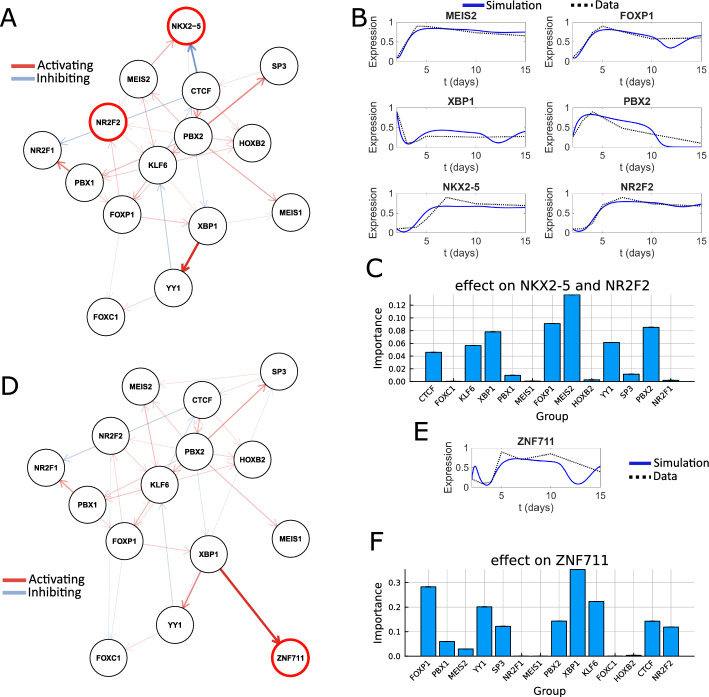
Dynamic model of epicardioid differentiation based on data from Meier et al. [23] **A**. Network topology of the model with one additional node, NKX2-5, selected using our data-driven approach for finding marker genes. **B**. Simulation results of important TFs in the model including NKX2-5 and NR2F2. Blue lines denote simulations, while dashed lines denote experimental data. We see that the simulation fits the experimental data well. **C**. Our model identifies MEIS2, FOXP1 and PBX2 to be the most important TFs to regulate NKX2-5 and NR2F2 expression. **D**. Network topology of the model with one additional node: ZNF711, selected from prior knowledge based on [43]. **E.** Simulated ZNF711 expression against the experimental data. **F**. Our model identifies XBP1, FOXP1 and KLF6 to be the most important TFs to regulate ZNF711 expression

In the constructed TF network we identified various known relevant transcription factors in cardiac development (Fig. 4A). For example, homeobox genes (such as HOXB2) contribute to patterning of cardiac progenitor cells and formation of the great arteries [38]. The transcription factor KLF6 is known to play a role in cardiac fibrosis in mice [39]. YY1 is essential for the commitment from mesodermal precursors into cardiac progenitor cells, after which YY1 maintains the progenitor stage by regulating key transcription factors such as NKX2-5 [40]. Simulations of the model constructed using DANSE mimic the experimental data (Fig. 4B).

To identify key TFs for genes of interest, we showcase the data-driven option and the prior knowledge option of the DANSE pipeline for selecting marker TFs, after which novel potential regulators can be found. When applying the data-driven option, NKX2-5 and NR2F2 were selected as marker TFs based on the ANANSE influence score going from day 2 to day 7, 10, and 15, as we are interested in drivers for markers of the later differentiation stages. From a biological perspective, these markers selected based on the data have clear roles within epicardioid differentiation. NKX2-5 plays important regulatory role in cardiac developmental program. It is a well-known marker of cardiac precursor cells and is involved in differentiation, atrial compartmentalization and conduction system formation [41]. The other selected marker gene, NR2F2, also known as COUP-TFII, is an atrial cardiomyocyte marker[42]. As only NR2F2 was included according to the top connections in the original model, we added NKX2-5 during the construction of the network. To do so, we add the two most likely activating and two most likely inhibiting connections to NKX2-5 to our TF network. These most likely connections are identified by the highest interaction score over all measured time points. We then asked which genes are most likely to affect NR2F2 and NKX2-5 activity.

Our model identified MEIS2, FOXP1 and PBX2 as potential regulators of NKX2-5 and NR2F2 during epicardioid differentiation. Based on our model, all three of these TFs should be upregulated to increase the expression of NKX2-5 and NR2F2 (Figure B7). In literature, PBX proteins have been found to be required for the activation of cardiac muscle differentiation in zebra fish embryos [44]. The work of [45] indicates the loss of PBX causes down-regulation of NKX2-5. FOXP1 is known to promote cardiomyocyte proliferation in early development, and has a known relation to NKX2-5 [46]. On the other hand, proteomic analysis has shown that FOXP proteins interact with NR2F2 on the protein level during cortical development [47]. These facts make FOXP1 a promising target. The transcription factor MEIS2 has been shown to be essential for cardiac neural crest development, and is known to promote cardiac conduction in mice [48, 49]. It is closely related to MEIS1 which shares overlapping binding sites with NKX2-5 on cardiac gene enhancers during differentiation [50]. Though evidence of a direct interaction between MEIS2 and NKX2-5 is lacking, our model suggests MEIS2 could be a potential driver of differentiation towards epicardioid. These results show the capability of DANSE to recover both known drivers of cardiac differentiation, as well as potential targets for which the role in cardiac differentiation is not as well-described in current literature.

When looking at the most important TF calculated from different choices for the amount of activating and inhibiting connections, we found that the three identified TFs are also inferred to be important regulators of NKX2-5 and NR2F2 for other choices of the number of activating and inhibiting connections. Even though the most important TF does change depending on the number of connections in the model, FOXP1 is recovered frequently as a TF that potentially regulates NKX2-5 and NR2F2 (Figure A5). This result shows some robustness of our method to recover the most important TF from the model.

In addition to the data-driven approach, we illustrate how to use our pipeline when the selection of marker genes is purely driven by prior knowledge. In particular, ZNF711 has recently been identified as a safeguard for cardiomyocyte commitment [43], and it is therefore interesting to ask which TFs may regulate ZNF711 expression. To investigate this, we added ZNF711 to the data-driven TF network following the same procedure as when adding NKX2-5. We then asked which TFs in the data-driven network are most likely to influence ZNF711 expression. We identified XBP1, FOXP1, and KLF6 as potential regulators of ZNF711. From these TFs, XBP1 is consistently found to be a regulator of ZNF711 for networks close to the considered network configuration. FOXP1 is also recovered several times as the second most important TF for networks with similar numbers of activating and inhibiting connections (Figure A6). To increase ZNF711 expression, our model suggests upregulation for all three identified drivers (Figure B7). Especially interesting is the role of FOXP1, as this TF is predicted to regulate both ZNF711 and NR2F2 in our computational models. While we could not find evidence of the effects of FOXP1 on ZNF711 expression in literature, this identifies FOXP1 as a promising target for optimising differentiation experiments toward epicardioid cells.

## Discussion

In this work, we present DANSE, a pipeline that can be used to construct a dynamic model based on time-series multi-omics data without explicitly providing prior knowledge-based input such as an initial gene regulatory network. We have illustrated the use of this pipeline by applying it to bulk data collected during a chondrogenesis experiment and single-cell data collected during a cardiogenesis experiment [21, 23]. The constructed TF networks contain transcription factors known to be important for both processes, and the resulting dynamic models mimic experimental gene expression data (Figs. 3 and 4). Subsequently, we have shown that our pipeline leads to the identification of interesting targets for upregulating or downregulating gene expression. For example, our chondrogenesis model suggests upregulating SREBF1 expression, which is not a well-known factor involved in this process. On the other hand, our epicardioid differentiation model suggests a potential role for FOXP1 in positively influencing ZNF711 expression. These roles could be further validated in wet-lab experiments. Our method of defining important transcription factors looks beyond the local role of individual nodes, instead focusing on the role of transcription factors within the full TF network. In some cases, the important transcription factors identified have only indirect effects on the selected target genes, instead modulating the expression of the target genes through the available TF network. While ATAC-seq data is always required, the pipeline does not depend on the format of the gene expression data. In this study, we have shown the application of our pipeline to the RNA-seq data available for the epicardioid differentiation case and to the microarray data available for the chondrogenesis case.

Beyond confirming known regulators, DANSE serves as a hypothesis-generating tool for biological discovery. It enables researchers to ask targeted, mechanistic questions that are central to understanding cell fate decisions. For example, based on the constructed model, it is possible to investigate which transcription factors drive a cell fate transition over time or what upstream regulators must be active to induce these key drivers. The pipeline also supports in silico perturbation experiments predicting the effects of knockdown or upregulation experiments (Figure B7). This allows biologists to explore how changes in TF expression affect the network as a whole, which may be experimentally validated using tools like CRISPR-Cas. Through the dynamic nature of the constructed computational models, our pipeline goes beyond what is possible with conventional methods such as pathway analysis [51, 52]. By translating multi-omics data into interpretable, dynamic models, DANSE creates a bridge between complex data and testable biological hypotheses and ultimately, a path toward predictive and programmable biology.

In recent years, the advent of single-cell sequencing has led to a drastic increase in detail of generated data. Pseudo-time trajectory inference is a method commonly used to describe temporal processes based on single-cell sequencing data [53, 54]. Through the resulting pseudo-time trajectory, branching points and other interesting features can be explored. These methods work well to describe the data, but do not directly provide mechanistic insights. While our pipeline is built for and tested on bulk data (as demonstrated using the chondrogenesis case in Fig. 3), it is also applicable to single-cell data, as shown in the application to the epicardioid differentiation experiment [23] (Fig. 4). Here, we have done so by looking at semi-bulk data for each time point, but one could follow a different, more detailed approach that does not abstract away cell heterogeneity. After an appropriate clustering of the single-cell data, TF networks can be constructed for each individual cluster using ANANSE [13]. Pseudotime inference methods such as slingshot or monocle3 can be used to infer the temporal ordering of these clusters, and can be used to infer branching points in the data so that individual trajectories of clusters can be constructed [55, 56]. Subsequently, the procedure described in this work can be used to select key TFs from each cluster and build a full TF network. If multiple branching trajectories are available, the network can be constructed based on only the clusters along one of these trajectories, but can also be constructed based on the alternative branches. The ODE model posed on this network can then be fitted to pseudo-time trajectories, finally resulting in a mechanistic model describing single-cell data. If more time points are desired for the model fitting procedure, a subclustering of the original clusters can be used to increase the granularity of the data. However, it is necessary to keep these subclusters from shrinking too much, as the data will become too noisy. As an alternative to running ANANSE, the scANANSE pipeline [13] can also be used to infer ANANSE networks based on single cell data. In addition to a streamlined pipeline to apply ANANSE to single-cell data, scANANSE can predict repressive TFs based on the comparison of motif z-scores between clusters [13].

To run the DANSE pipeline, an initial choice of the number of activating and inhibiting interactions as well as the desired number of TFs for the network is required. In a practical use case, this means that the pipeline should be run multiple times to account for the effects of these hyperparameter choices. For the choice of TFs in our two examples, we have investigated ANANSE influence plots to see which TFs are included in the network topology (see Figures A1, D10 and D12). Based on these plots, a choice for the cut-off value for the number of TFs was made. Next, a user should choose the number of activating and inhibiting connections. Here, we suggest running DANSE multiple times for different choices of the number of activating and inhibiting connections, and evaluating model results for the found configurations. For the two cases discussed in this paper, we have looked at the final value of the loss function as a metric for the performance of the model (Figures A2 and A4). If the loss drops significantly when adding connections, it is likely that adding these connections is a good decision. More connections should be added to the model if the resulting models do not match trends in the experimental gene expression data. Fewer connections should be selected if the model shows biologically unfeasible behaviour (for example, oscillations in a known non-oscillating system) or very different mechanisms for each run fit of the model are found. Hence, these parameter sweeps are instrumental to the construction of an informative computational model and verifying how robust the recovered mechanism is to these hyperparameter choices. We note that it is not possible to automate these hyperparameter choices, since different input data lead to models with different characteristics. Therefore, we emphasise that these are necessary modelling choices, while DANSE provides a tool to convert these modelling choices to a dynamic model along with a first analysis of the resulting model. In this way, DANSE should be seen as part of a modelling cycle, where the model is iteratively updated until a prediction is made, leading to new data which can be incorporated in the model. An overview of the workflow using DANSE is shown in Fig. 1, illustrating this modelling cycle.

When selecting the hyperparameters necessary to run the DANSE pipeline, a concern is whether the model is overfitting to the data. We have found that while the top identified key TF does not change with every added or removed connection, the output of our pipeline varies when considering wider ranges of hyperparameter choices. When considering a large range of hyperparameter choices for our two use cases, we find that many of the top identified TFs are robustly found to be important for many of the alternative choices (Figures A3, A5 and A6). This is in line with recent theoretical work, where biological networks were found to exhibit robust dynamics even when nodes and edges are removed [57]. Our approach of finding a minimal network that exhibits a good fit to the data should then be seen as finding the smallest network configuration that leads to the desired dynamics. Still, the recovery of many potential models with differing underlying mechanisms reinforces the need for the setup of a modelling cycle, where the alternative hypotheses posed by these different computational models can be validated in a wet-lab setting. We note that conventional methods of checking overfitting are not readily applicable to the models generated using DANSE, as only the small TF network and gene expression data at sparse time points for these TFs are used to fit the model. Therefore, leaving out one data point as a “test set” is possible, but will result in removing so much information from both the network construction and time series data used for fitting the model that it becomes difficult to compare results. We have therefore chosen to focus only on model choices and hyperparameters to evaluate the performance of models constructed using DANSE.

The loss function currently used to optimise parameters of the ODE system optimises the squared error between data and simulation, while promoting sparsity through the addition of an L1 loss term. In some cases, this procedure led to a fit to the data where the resulting simulations had undesirable properties, such as a slow convergence to a steady state or (damped) oscillatory behaviour (see, for example, the fit for ZNF711 in Fig. 4E). To remedy this, the loss function could be adapted if prior knowledge on the biological system is available. For example, if validated connections are part of the underlying network, one could leave out these interactions from the L1 term. Similarly, if the system is known to converge to a steady-state within the simulated time, a term could be added to penalise the value of the right-hand side of the ODE evaluated at the desired steady states.

Next to the network structure, one can consider the sign of the interactions. In the current approach, the interaction score is allocated a sign that reflects the predicted effect of a TF on the collective enhancer regions connected to a specific target gene. This interaction is activating or inhibiting, which means this model allows a TF to be both a repressor or activator in the same network, depending on which enhancers it is binding. From a mechanistic point of view, this is a simplification. With some exceptions, for example nuclear hormone receptors that act as an activator or repressor depending on the presence of the nuclear hormone ligand [58], transcription factors tend to be either activators or repressors and do not have both functionalities. However, the consequences of their perturbation can be highly complex, due to both indirect effects of the regulatory circuitry [59], and enhancer grammar and syntax [60]. Integrating complex regulatory input at the enhancer level can accommodate highly complex temporal or spatial expression patterns without invoking a variable activity of TFs [61]. On the other hand, enhancer grammar may involve a different number, orientation and spacing of motifs that can be bound by TFs, causing individual TFs to produce a context-dependent effect on a regulatory region. In DANSE, our approach is to model the TF’s effect on the regulatory regions in the context of that specific target gene - of which the expression is also taken along to calculate the interaction score ($E_j$) - as a simplified model of the context-dependent effects of TFs on target genes.

A limitation of the DANSE pipeline is that we assume that connections as well as optimised connection strengths between TFs are static throughout a modelled process. This may not be the case in reality, where responses of cells to specific stimuli vary in different cell types. A possible adjustment to the pipeline could be made, where a network is inferred for each individual time point. Computational models based on these individual networks could be linked together to form a multi-scale model of a longer temporal process.

In this work, we have focussed on the use of ANANSE to identify core transcription factor networks and influential transcription factors through the comparison of these networks [11]. However, other network inference methods based on multi-omics data are available [12, 62, 63]. Such a comparison to other inference methods would require a procedure to select a relevant sub-network including important transcription factors, where we have used ANANSE’s influence score to select TFs from the data. It would be interesting to consider how the use of different inference methods affects resulting dynamic models.

Looking at the interpretation of the models built using DANSE, mechanistic modelling of (semi-)bulk data has restrictions resulting from the lack of granularity per measured time point. Therefore, mechanistic models built using our pipeline describe changes in gene expression of one population of cells over different time points, but cannot track multiple cell types, since the model is fit to one time series. This is a limiting factor in the case where single cell data is considered in which cells split into two trajectories. With our current implementation of the DANSE pipeline, it is only possible to construct a model for each of the trajectories individually. However, a model describing all possible trajectories may be desired. To achieve the identification of such a model, a potential approach is to redefine the procedure used for fitting the model to the data by simulating a population of cells, and using methods from optimal transport to compute the goodness of fit [64]. This probabilistic view of the fitting procedure may also help to overcome the fact that our current method does not take noise into account.

## Conclusion

DANSE allows to go beyond static descriptions of gene regulation, and offers a framework for building dynamic, interpretable, models directly from multi-omics data. By integrating network inference based on transcription factor binding sites and transcriptomics with dynamic modelling, DANSE allows to go from observational data to mechanistic hypotheses in a data-driven manner. The ability to identify a minimal network of key regulators of cellular processes makes it a novel and powerful tool for uncovering both known and novel drivers of cell fate decisions. In this paper, we have shown these capabilities in two case studies for chondrogenesis and epicardioid differentiation. DANSE has the potential to transform the way we analyse time-series data in tissue development, regeneration and disease progression in a data-driven and objective manner.

## Supplementary Information

Supplementary Material 1.

## Data Availability

All code for the DANSE pipeline is available on https://github.com/LJansenKlomp/DANSE. The datasets analysed during this study are available in the NCBI Gene Expression Omnibus repository. The data for the chondrogenesis experiment described in [21] can be accessed using accession numbers GEO: GSE96036, GSE109172 and GSE132532. The data for the epicardioid differentiation experiment described in [23] can be accessed using accession number GEO: GSE196516. We have used BioRender to create Fig. 1. The license for this figure can be found at https://BioRender.com/44wye8n.

## References

[1] Bar-Joseph Z, Gitter A, Simon I. Studying and modelling dynamic biological processes using time-series gene expression data. Nat Rev Genet. 2012;13(8):552–64. 22805708 10.1038/nrg3244

[2] Tripto NI, Kabir M, Bayzid MS, Rahman A. Evaluation of classification and forecasting methods on time series gene expression data. PLoS ONE. 2020;15(11):0241686. 10.1371/journal.pone.0241686 PMC7647064 33156855

[3] Jashnsaz H, Fox ZR, Munsky B, Neuert G. Building predictive signaling models by perturbing yeast cells with time-varying stimulations resulting in distinct signaling responses. Star Protoc. 2021;2(3):100660. 34286292 10.1016/j.xpro.2021.100660 PMC8273411

[4] Rukhlenko OS, Kholodenko BN. Modeling the nonlinear dynamics of intracellular signaling networks. Bio-Protoc. 2021;11(14):4089–4089. 10.21769/BioProtoc.4089 PMC8329461 34395728

[5] Barbuti R, Gori R, Milazzo P, Nasti L. A survey of gene regulatory networks modelling methods: from differential equations, to Boolean and qualitative bioinspired models. J Membr Comput. 2020;2(3):207–26.

[6] Kim D, Tran A, Kim HJ, Lin Y, Yang JYH, Yang P. Gene regulatory network reconstruction: harnessing the power of single-cell multi-omic data. NPJ Syst Biol Appl. 2023;9(1):51. 37857632 10.1038/s41540-023-00312-6 PMC10587078

[7] Nguyen H, Tran D, Tran B, Pehlivan B, Nguyen T. A comprehensive survey of regulatory network inference methods using single cell RNA sequencing data. Brief Bioinform. 2021;22(3):190. 10.1093/bib/bbaa190 PMC8138892 34020546

[8] Pratapa A, Jalihal AP, Law JN, Bharadwaj A, Murali T. Benchmarking algorithms for gene regulatory network inference from single-cell transcriptomic data. Nat Methods. 2020;17(2):147–54. 31907445 10.1038/s41592-019-0690-6 PMC7098173

[9] Huynh-Thu VA, Irrthum A, Wehenkel L, Geurts P. Inferring regulatory networks from expression data using tree-based methods. PLoS ONE. 2010;5(9):12776. 10.1371/journal.pone.0012776 PMC2946910 20927193

[10] Margolin AA, Nemenman I, Basso K, Wiggins C, Stolovitzky G, Favera RD, Califano A. ARACNE: an algorithm for the reconstruction of gene regulatory networks in a mammalian cellular context. In: BMC Bioinformatics, 2006:7;1–15 . Springer. 10.1186/1471-2105-7-S1-S7 PMC1810318 16723010

[11] Xu Q, Georgiou G, Frölich S, Sande M, Veenstra GJC, Zhou H, et al. ANANSE: an enhancer network-based computational approach for predicting key transcription factors in cell fate determination. Nucleic Acids Res. 2021;49(14):7966–85. 34244796 10.1093/nar/gkab598 PMC8373078

[12] Kamal A, Arnold C, Claringbould A, Moussa R, Servaas NH, Kholmatov M, et al. GRaNIE and GRaNPA: inference and evaluation of enhancer-mediated gene regulatory networks. Mol Syst Biol. 2023;19(6):11627. 10.15252/msb.202311627 PMC10258561 37073532

[13] Smits JG, Arts JA, Frölich S, Snabel RR, Heuts BM, Martens JH, et al. scANANSE gene regulatory network and motif analysis of single-cell clusters. F1000Research. 2023;12:243. 38116584 10.12688/f1000research.130530.1 PMC10728588

[14] Bravo González-Blas C, De Winter S, Hulselmans G, Hecker N, Matetovici I, Christiaens V, et al. SCENIC+: single-cell multiomic inference of enhancers and gene regulatory networks. Nat Methods. 2023;20(9):1355–67. 37443338 10.1038/s41592-023-01938-4 PMC10482700

[15] Matsumoto H, Kiryu H, Furusawa C, Ko MS, Ko SB, Gouda N, et al. SCODE: an efficient regulatory network inference algorithm from single-cell RNA-Seq during differentiation. Bioinformatics. 2017;33(15):2314–21. 28379368 10.1093/bioinformatics/btx194 PMC5860123

[16] Chen X, Li M, Zheng R, Wu F-X, Wang J. D3GRN: a data driven dynamic network construction method to infer gene regulatory networks. BMC Genom. 2019;20:1–8. 10.1186/s12864-019-6298-5 PMC6933629 31881937

[17] Huynh-Thu VA, Geurts P. dynGENIE3: dynamical GENIE3 for the inference of gene networks from time series expression data. Sci Rep. 2018;8(1):3384. 29467401 10.1038/s41598-018-21715-0 PMC5821733

[18] Hossain I, Fanfani V, Quackenbush J, Burkholz R. Biologically informed neuralODEs for genome-wide regulatory dynamics. Research Square;2023. 10.1186/s13059-024-03264-0 PMC11106922 38773638

[19] Zhou JX, Brusch L, Huang S. Predicting pancreas cell fate decisions and reprogramming with a hierarchical multi-attractor model. PLoS ONE. 2011;6(3):14752. 10.1371/journal.pone.0014752 PMC3056652 21423725

[20] Mirams GR, Byrne HM, King JR. A multiple timescale analysis of a mathematical model of the Wnt/-catenin signalling pathway. J Math Biol. 2010;60:131–60. 19288106 10.1007/s00285-009-0262-y

[21] Kawata M, Mori D, Kanke K, Hojo H, Ohba S, Chung U-I, et al. Simple and robust differentiation of human pluripotent stem cells toward chondrocytes by two small-molecule compounds. Stem Cell Rep. 2019;13(3):530–44. 10.1016/j.stemcr.2019.07.012 PMC6739881 31402337

[22] Loh KM, Chen A, Koh PW, Deng TZ, Sinha R, Tsai JM, et al. Mapping the pairwise choices leading from pluripotency to human bone, heart, and other mesoderm cell types. Cell. 2016;166(2):451–67. 27419872 10.1016/j.cell.2016.06.011 PMC5474394

[23] Meier AB, Zawada D, De Angelis MT, Martens LD, Santamaria G, Zengerle S, et al. Epicardioid single-cell genomics uncovers principles of human epicardium biology in heart development and disease. Nat Biotechnol. 2023;41(12):1787–800. 37012447 10.1038/s41587-023-01718-7 PMC10713454

[24] Kang X, Hajek B, Hanzawa Y. From graph topology to ODE models for gene regulatory networks. PLoS ONE. 2020;15(6):0235070. 10.1371/journal.pone.0235070 PMC7326199 32603340

[25] Polynikis A, Hogan S, Di Bernardo M. Comparing different ODE modelling approaches for gene regulatory networks. J Theor Biol. 2009;261(4):511–30. 19665034 10.1016/j.jtbi.2009.07.040

[26] Kingma DP, Ba J. Adam: A method for stochastic optimization;2014. arXiv:1412.6980.

[27] Ritchie ME, Phipson B, Wu D, Hu Y, Law CW, Shi W, et al. limma powers differential expression analyses for RNA-sequencing and microarray studies. Nucleic Acids Res. 2015;43(7):47–47. 10.1093/nar/gkv007 PMC4402510 25605792

[28] Hao Y, Stuart T, Kowalski MH, Choudhary S, Hoffman P, Hartman A, et al. Dictionary learning for integrative, multimodal and scalable single-cell analysis. Nat Biotechnol. 2023. 10.1038/s41587-023-01767-y. 37231261 10.1038/s41587-023-01767-y PMC10928517

[29] Stuart T, Srivastava A, Madad S, Lareau C, Satija R. Single-cell chromatin state analysis with Signac. Nat Methods. 2021. 10.1038/s41592-021-01282-5. 34725479 10.1038/s41592-021-01282-5 PMC9255697

[30] Dyer SC, Austine-Orimoloye O, Azov AG, Barba M, Barnes I, Barrera-Enriquez VP, et al. Ensembl 2025. Nucleic Acids Res. 2025;53(D1):948–57. 10.1093/nar/gkae1071 PMC11701638 39656687

[31] Lu Y, Yin Y, Kats ER, Sun J, Liu S, Lin H, Estrogen receptor- loss accelerates cartilage degradation through CLEC3B-mediated chondrocyte hypertrophy and inflammation. Osteoarthritis and Cartilage;2025. 10.1016/j.joca.2025.09.001 40915381

[32] Kim Y-I, No Lee J, Bhandari S, Nam I-K, Yoo K-W, Kim S-J, et al. Cartilage development requires the function of Estrogen-related receptor alpha that directly regulates sox9 expression in zebrafish. Sci Rep. 2015;5(1):18011. 26657540 10.1038/srep18011 PMC4675082

[33] Lefebvre V, Angelozzi M, Haseeb A. SOX9 in cartilage development and disease. Curr Opin Cell Biol. 2019;61:39–47. 31382142 10.1016/j.ceb.2019.07.008 PMC6956855

[34] Lefebvre V, Dvir-Ginzberg M. SOX9 and the many facets of its regulation in the chondrocyte lineage. Connect Tissue Res. 2017;58(1):2–14. 27128146 10.1080/03008207.2016.1183667 PMC5287363

[35] Liu C-F, Lefebvre V. The transcription factors SOX9 and SOX5/SOX6 cooperate genome-wide through super-enhancers to drive chondrogenesis. Nucleic Acids Res. 2015;43(17):8183–203. 26150426 10.1093/nar/gkv688 PMC4787819

[36] Green JD, Tollemar V, Dougherty M, Yan Z, Yin L, Ye J, et al. Multifaceted signaling regulators of chondrogenesis: Implications in cartilage regeneration and tissue engineering. Genes & diseases. 2015;2(4):307–27. 26835506 10.1016/j.gendis.2015.09.003 PMC4730920

[37] Liu F, Wang X, Zheng B, Li D, Chen C, Lee I-S, et al. USF2 enhances the osteogenic differentiation of PDLCs by promoting ATF4 transcriptional activities. J Periodontal Res. 2020;55(1):68–76. 31448831 10.1111/jre.12689

[38] Roux M, Zaffran S. Hox genes in cardiovascular development and diseases. J Dev Biol. 2016;4(2):14. 29615581 10.3390/jdb4020014 PMC5831787

[39] Sawaki D, Hou L, Tomida S, Sun J, Zhan H, Aizawa K, et al. Modulation of cardiac fibrosis by Krüppel-like factor 6 through transcriptional control of thrombospondin 4 in cardiomyocytes. Cardiovasc Res. 2015;107(4):420–30. 25987545 10.1093/cvr/cvv155 PMC4540142

[40] Gregoire S, Li G, Sturzu AC, Schwartz RJ, Wu SM. YY1 expression is sufficient for the maintenance of cardiac progenitor cell state. Stem Cells. 2017;35(8):1913–23. 28580685 10.1002/stem.2646 PMC6048588

[41] Cao C, Li L, Zhang Q, Li H, Wang Z, Wang A, et al. Nkx2. 5: a crucial regulator of cardiac development, regeneration and diseases. Front Cardiovasc Med. 2023;10:1270951. 38124890 10.3389/fcvm.2023.1270951 PMC10732152

[42] Pereira FA, Qiu Y, Zhou G, Tsai M-J, Tsai SY. The orphan nuclear receptor COUP-TFII is required for angiogenesis and heart development. Genes Dev. 1999;13(8):1037–49. 10215630 10.1101/gad.13.8.1037 PMC316637

[43] Snabel RR, Cofiño-Fabrés C, Baltissen M, Schwach V, Passier R, Veenstra GJC. Cardiac differentiation roadmap for analysis of plasticity and balanced lineage commitment. Stem Cell Rep. 2025;20(3):102422. 10.1016/j.stemcr.2025.102422. 10.1016/j.stemcr.2025.102422 PMC11960529 40020683

[44] Maves L, Tyler A, Moens CB, Tapscott SJ. Pbx acts with Hand2 in early myocardial differentiation. Dev Biol. 2009;333(2):409–18. 19607825 10.1016/j.ydbio.2009.07.004 PMC2752274

[45] Koss M, Bolze A, Brendolan A, Saggese M, Capellini TD, Bojilova E, et al. Congenital asplenia in mice and humans with mutations in a Pbx/Nkx2-5/p15 module. Dev Cell. 2012;22(5):913–26. 22560297 10.1016/j.devcel.2012.02.009 PMC3356505

[46] Zhang Y, Li S, Yuan L, Tian Y, Weidenfeld J, Yang J, et al. Foxp1 coordinates cardiomyocyte proliferation through both cell-autonomous and nonautonomous mechanisms. Genes & development. 2010;24(16):1746–57. 20713518 10.1101/gad.1929210 PMC2922503

[47] Estruch SB, Graham SA, Quevedo M, Vino A, Dekkers DH, Deriziotis P, et al. Proteomic analysis of FOXP proteins reveals interactions between cortical transcription factors associated with neurodevelopmental disorders. Hum Mol Genet. 2018;27(7):1212–27. 29365100 10.1093/hmg/ddy035

[48] Machon O, Masek J, Machonova O, Krauss S, Kozmik Z. Meis2 is essential for cranial and cardiac neural crest development. BMC Dev Biol. 2015;15:1–16. 26545946 10.1186/s12861-015-0093-6 PMC4636814

[49] Muñoz-Martín N, Simon-Chica A, Díaz-Díaz C, Cadenas V, Temiño S, Esteban I, et al. Meis transcription factors regulate cardiac conduction system development and adult function. Cardiovasc Res. 2025;121(2):311–23. 39691060 10.1093/cvr/cvae258 PMC12012448

[50] Dupays L, Shang C, Wilson R, Kotecha S, Wood S, Towers N, et al. Sequential binding of MEIS1 and NKX2-5 on the Popdc2 gene: a mechanism for spatiotemporal regulation of enhancers during cardiogenesis. Cell Rep. 2015;13(1):183–95. 26411676 10.1016/j.celrep.2015.08.065 PMC4597108

[51] Ashburner M, Ball CA, Blake JA, Botstein D, Butler H, Cherry JM, et al. Gene ontology: tool for the unification of biology. Nat Genet. 2000;25(1):25–9. 10802651 10.1038/75556 PMC3037419

[52] Kanehisa M, Goto S. KEGG: kyoto encyclopedia of genes and genomes. Nucleic Acids Res. 2000;28(1):27–30. 10592173 10.1093/nar/28.1.27 PMC102409

[53] Saelens W, Cannoodt R, Todorov H, Saeys Y. A comparison of single-cell trajectory inference methods. Nat Biotechnol. 2019;37(5):547–54. 30936559 10.1038/s41587-019-0071-9

[54] Deconinck L, Cannoodt R, Saelens W, Deplancke B, Saeys Y. Recent advances in trajectory inference from single-cell omics data. Curr Opin Syst Biol. 2021;27:100344.

[55] Street K, Risso D, Fletcher RB, Das D, Ngai J, Yosef N, et al. Slingshot: cell lineage and pseudotime inference for single-cell transcriptomics. BMC Genom. 2018;19(1):477. 10.1186/s12864-018-4772-0 PMC6007078 29914354

[56] Trapnell C, Cacchiarelli D, Grimsby J, Pokharel P, Li S, Morse M, et al. The dynamics and regulators of cell fate decisions are revealed by pseudotemporal ordering of single cells. Nat Biotechnol. 2014;32(4):381–6. 24658644 10.1038/nbt.2859 PMC4122333

[57] Tripathi S, Kessler DA, Levine H. Minimal frustration underlies the usefulness of incomplete regulatory network models in biology. Proc Natl Acad Sci. 2023;120(1):2216109120. 10.1073/pnas.2216109120 PMC9910462 36580597

[58] Le Maire A, Germain P, Bourguet W. Protein-protein interactions in the regulation of RAR–RXR heterodimers transcriptional activity. In: Methods in Enzymology, 2020:637;175–207. Elsevier. 10.1016/bs.mie.2020.02.007 32359645

[59] Alon U. Network motifs: theory and experimental approaches. Nat Rev Genet. 2007;8(6):450–61. 17510665 10.1038/nrg2102

[60] Jindal GA, Farley EK. Enhancer grammar in development, evolution, and disease: dependencies and interplay. Dev Cell. 2021;56(5):575–87. 33689769 10.1016/j.devcel.2021.02.016 PMC8462829

[61] Segal E, Raveh-Sadka T, Schroeder M, Unnerstall U, Gaul U. Predicting expression patterns from regulatory sequence in Drosophila segmentation. Nature. 2008;451(7178):535–40. 18172436 10.1038/nature06496

[62] Aibar S, González-Blas CB, Moerman T, Huynh-Thu VA, Imrichova H, Hulselmans G, et al. SCENIC: single-cell regulatory network inference and clustering. Nat Methods. 2017;14(11):1083–6. 28991892 10.1038/nmeth.4463 PMC5937676

[63] Badia-i-Mompel P, Wessels L, Müller-Dott S, Trimbour R, Ramirez Flores RO, Argelaguet R, et al. Gene regulatory network inference in the era of single-cell multi-omics. Nat Rev Genet. 2023;24(11):739–54. 37365273 10.1038/s41576-023-00618-5

[64] Tong A, Huang J, Wolf G, Van Dijk D, Krishnaswamy S. Trajectorynet: A dynamic optimal transport network for modeling cellular dynamics. In: International Conference on Machine Learning, pp. 2020;9526–9536 . PMLR. PMC8320749 34337419

