# Supplementary material for: DANSE: a pipeline for dynamic modelling of time-series multi-omics data
Source: BMC Bioinformatics. 2025 Dec 30;27:28. doi: 10.1186/s12859-025-06354-3 (PMC12859988; doi:10.1186/s12859-025-06354-3)
Supplement: Supplementary file 1 — Supplementary Material 1. [file 12859_2025_6354_MOESM1_ESM.pdf]

# Appendix A Details on parameter selection

Here, we provide additional details on the hyperparameter selection for the two models presented in this work. In this way, we provide the rationale behind our modelling choices, and provide meaningful pointers to users of the DANSE pipeline when selecting these hyperparameters for other biological systems.

## A.1 The chondrogenesis model

To construct a gene regulatory network, we first select the number of transcription factors that should be added to the network. Here, we selected the top 5 TFs per influence computation (example shown in Figure A1), and the top 12 nodes with highest average degree. It can be checked that well-known targets are not directly below the selection threshold in this phase. For this application of the pipeline, this did not seem to be the case.

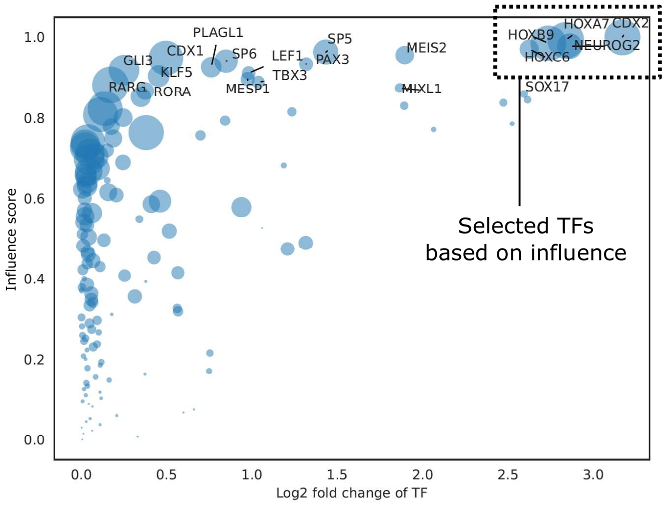


**Fig. A1**: *Influence plot for D0 → D2 in the The horizontal axis indicates fold change of expression level, whereas the vertical axis indicates change of the influence score, as determined using ANANSE [11]. The size of each dot indicates the expression level in the target cell population. The top 5 TFs (all selected TFs for the network from this influence plot) are indicated. The threshold of how many TFs are selected can be adjusted in DANSE, and desired TFs can be manually added.*

We then varied the size of the network to select our model structure. We first checked that the size of network was mostly constrained by the number of interactions, rather than the number of TFs selected. Since this was the case for this application of the pipeline, we chose to focus only on the number of interactions to assess the

effects of the network structure on the model inferred using DANSE. We ran the model parameter fit for networks of different size to find a minimal network that gives a good fitting result, based on the average squared loss value per node in the network. Doing so, we found that small networks with 70 activating connections or more resulted in a good fit. In addition, the goodness of fit decreases past 110 activating connections. We chose to focus on the cut-off at 70 activating connections to obtain a minimal network that recapitulates the data, and used the configuration with 40 inhibiting connections as it empirically provided the best fit in the grid search.

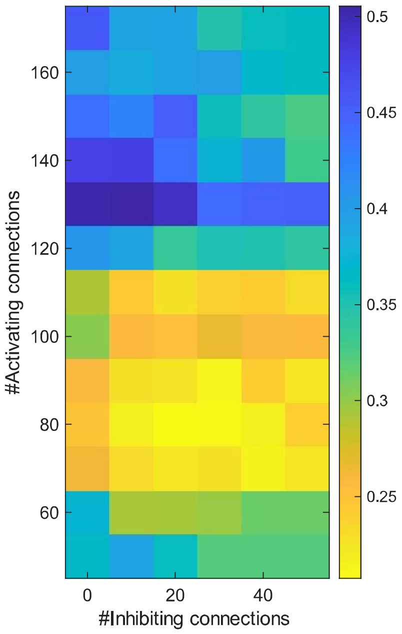


**Fig. A2**: *Average value of loss function over all nodes in chondrogenic differentiation model. The window between 70 activating connections and 110 activating connections where the loss is lower than for other hyperparameter choices is a key indicator for the choice of activating interactions when running DANSE.*

To assess the robustness of the results, we ran multiple fits for the selected configuration of 70 activating connections and 40 inhibiting connections. These 10 individual fits are used to assess the uncertainty of the importance scores for different TFs as presented in Figure 3C. Next to robustness within a single network structure, it is useful to look at nearby network configurations of different sizes. We recomputed the importance score for all TFs for each of the networks with different sizes constructed to select a TF network. This allowed us to assess the sensitivity of the importance score on the network size (Figure A3). This analysis showed that USF2 is consistently the most influential node, as it directly activates SOX9 and SOX5 in the model. The TFs FOXK2 and SREBF1 are often found to be the second most important TF.

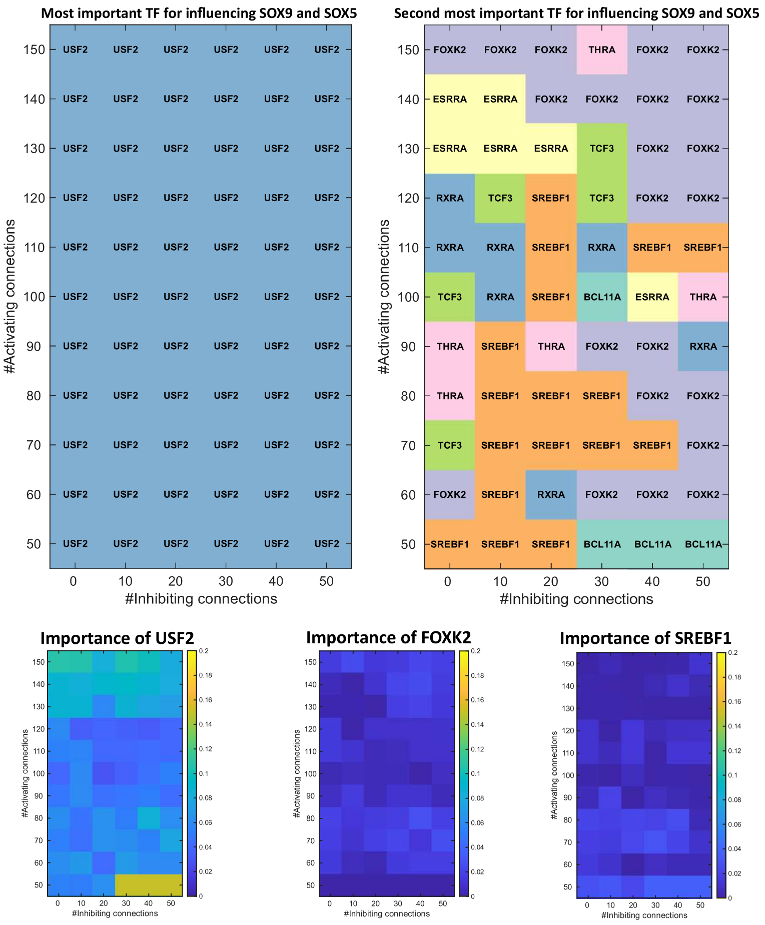


**Fig. A3**: The most important TF as identified by the dynamical model constructed using DANSE for the chondrogenesis data [21] for different choices of activating and inhibiting connections. These TFs are classified as important by their influence on SOX9 and SOX5 expression in the constructed computational model. Here, only one fit is considered per grid point, which may cause minor differences in results compared to the statistics over 10 fits as shown in Figure 3. We also show the second most important TF for influencing these markers. **top**. Overview of the most important and second most important TF influencing SOX9 and SOX5 as identified for different choices for the number of activating and inhibiting connections. **bottom**. Importance for influencing SOX9 and SOX5 as calculated for different TFs. The calculation is shown for a varying number of selected activating and inhibiting connections.

## A.2 The cardiogenesis model

We repeated the procedure outlined for the chondrogenesis model for the epicardioid differentiation model. Specifically, we first selected the top 5 TFs per influence computation, and selected the top 12 TFs with highest average degree in the core transcription factor networks. We then chose to again focus on the effects of the number of interactions on the resulting network.

We found similar results as for the chondrogenesis model when assessing the goodness of fit for different network sizes. Specifically, there is a clear cut-off around 130 activating connections, where the obtained loss is much lower for networks with more than 130 activating connections, and higher for smaller networks. We therefore selected 130 activating connections, and chose 20 inhibiting connections, as there is a drop in the final loss value going from 10 to 20 inhibiting connections, but no large benefits for selecting more inhibiting connections.

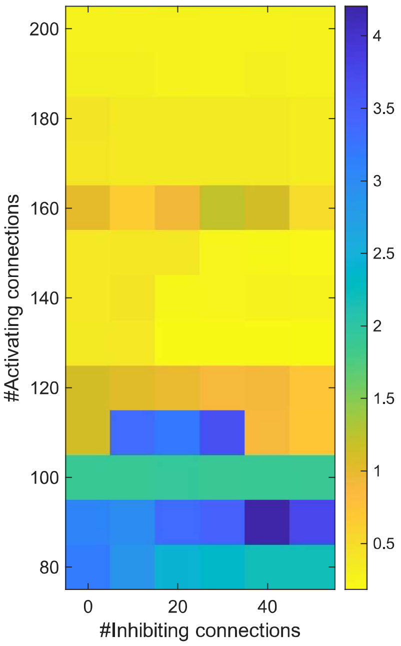


**Fig. A4**: *Average value of loss function over all nodes in epicardioid differentiation model.**left** Indication that model performs best with around 200 activating and 40 inhibiting connections. **right** Value over zoomed in grid, indicating 200 activating and 44 inhibiting connections to be the reasonable cut-off configuration*

We again assessed the robustness of the model by rerunning the fit for the selected number of connections to compute the SEM of the importance metric presented in Figure 4C,E. We also looked again at the influence of the number of interactions on the TFs identified to have the most importance. We see that FOXP1 has consistent

effects on HAND2, NR2F2 and ZNF711. However, we see here that some important TFs may be missed if an ensemble of networks is not considered. For example, PBX2 and NR2F1 are found to be important TFs in the network, but only for specific bands of the number of activating connections in the network.

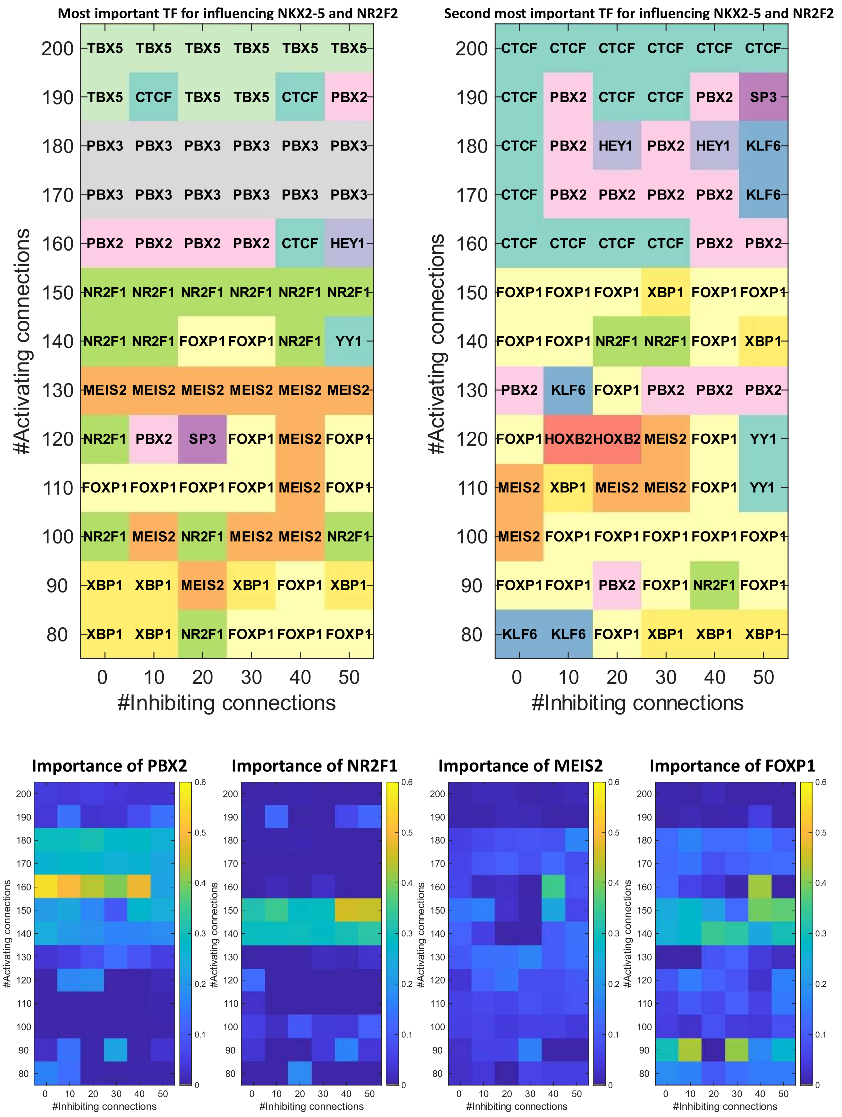


**Fig. A5**: The most important TFs as identified by the dynamical model constructed using DANSE for the epicardioid differentiation data [23] for different choices of activating and inhibiting connections. These TFs are classified as important by their influence on NKX2-5 and NR2F2 expression in the constructed computational model. Here, only one fit is considered per grid point, which may cause minor differences in results compared to the statistics over 10 fits as shown in Figure 4. **top**. Overview of the most important and second most important TF influencing NKX2-5 and NR2F2 as identified for different choices for the number of activating and inhibiting connections. **bottom**. Importance for influencing NKX2-5 and NR2F2 as calculated for different TFs. The calculation is shown for a varying number of selected activating and inhibiting connections.

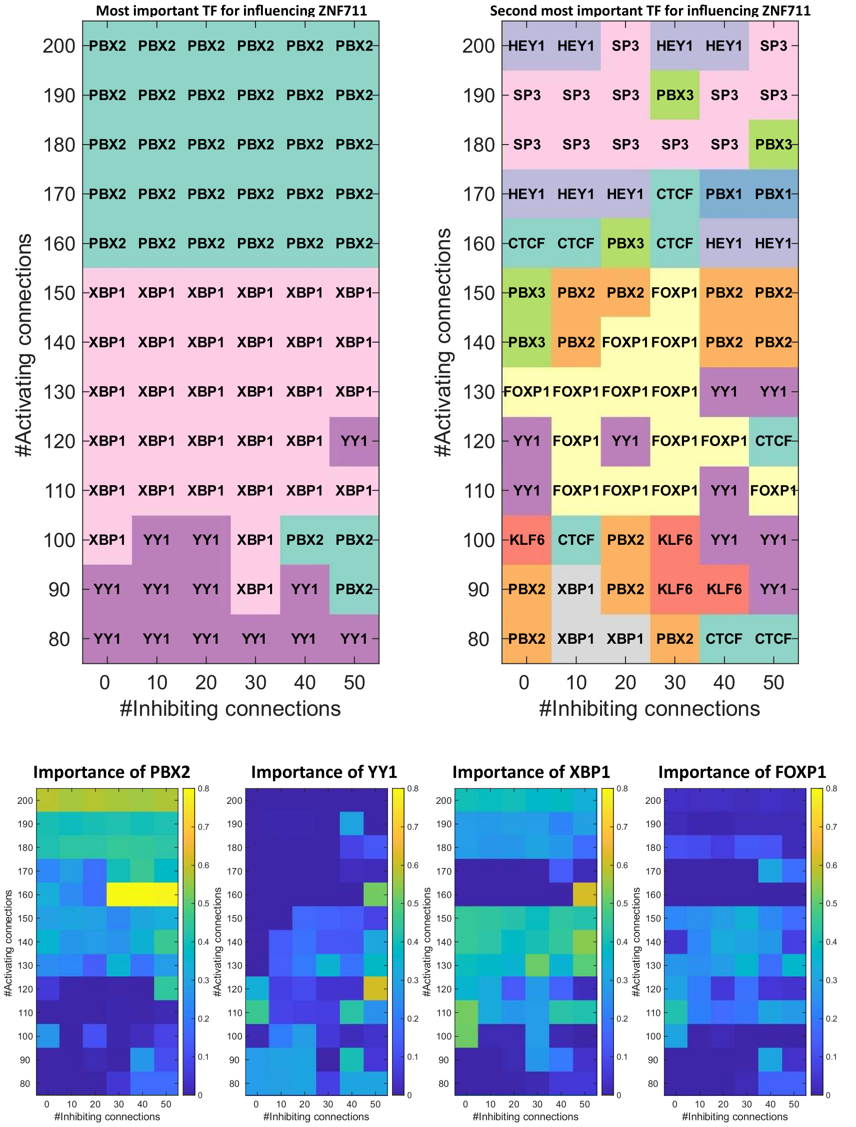


**Fig. A6**: The most important TFs as identified by the dynamical model constructed using DANSE for the epicardioid differentiation data [23] for different choices of activating and inhibiting connections. These TFs are classified as important by their influence on ZNF711 expression in the constructed computational model. Here, only one fit is considered per grid point, which may cause minor differences in results compared to the statistics over 10 fits as shown in Figure 4. **top**. Overview of the most important and second most important TF influencing ZNF711 as identified for different choices for the number of activating and inhibiting connections. **bottom**. Importance for influencing ZNF711 as calculated for different TFs. The calculation is shown for a varying number of selected activating and inhibiting connections.

# Appendix B Effects of knockdown/overexpression of driver TFs on identified key transcription factors

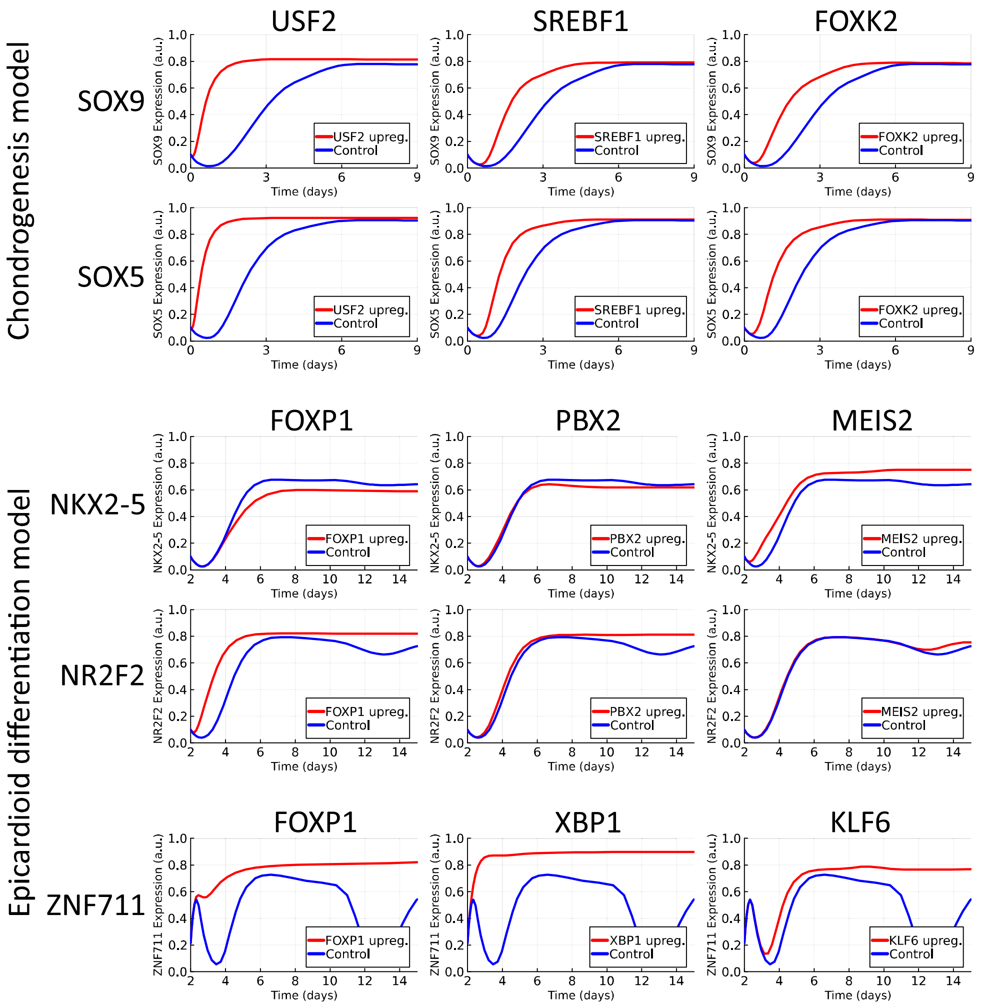


**Fig. B7**: Simulated knockdown/upregulation experiments for the most important TFs identified in Figures 3 and 4, showing the effects of perturbation of the identified drivers on the considered target TFs. For each upregulation experiment, we set $p_{\text{act}} = 3$ in System 3, whereas for each knockdown experiment, we set $p_{\text{inh}} = 3$, and we compare the simulation with knockdown/upregulation to the original simulation of the model. In the simulations shown, the blue line shows the original simulation without knockdown or upregulation, and the red line shows the simulation with knockdown or upregulation.

# Appendix C Effects of stabilising gene expression before fitting

We here test the influence of the applied scaling procedure of the data in the case that gene expression is nearly constant over the measured time period. In this case, noise is amplified through the scaling of the data, and we therefore ask what the effect of stabilising these genes is on the resulting importance measures computed using DANSE. We have tested this procedure on the chondrogenesis model, where 6 of the 13 genes had low variance in gene expression (Figure C8, top left). We iteratively changed the expression of these genes to:

$$D_i(t) = \frac{1}{2} + \frac{\mathrm{Var}(D_i)}{\max_j \mathrm{Var}(D_j)} \left( D_i(t) - \frac{1}{2} \right). \tag{C1}$$

Here, $D_i(t)$ denotes the gene expression data for TF $i$ at time $t$, whereas $D_i$ denotes the raw gene expression data of TF $i$ along the full time series data. This procedure centers the gene expression around $\frac{1}{2}$, with some small deviations still possible based on the variance in gene expression. We subsequently fitted models based on the adapted data, and looked at the effects on the importance score computed using DANSE.

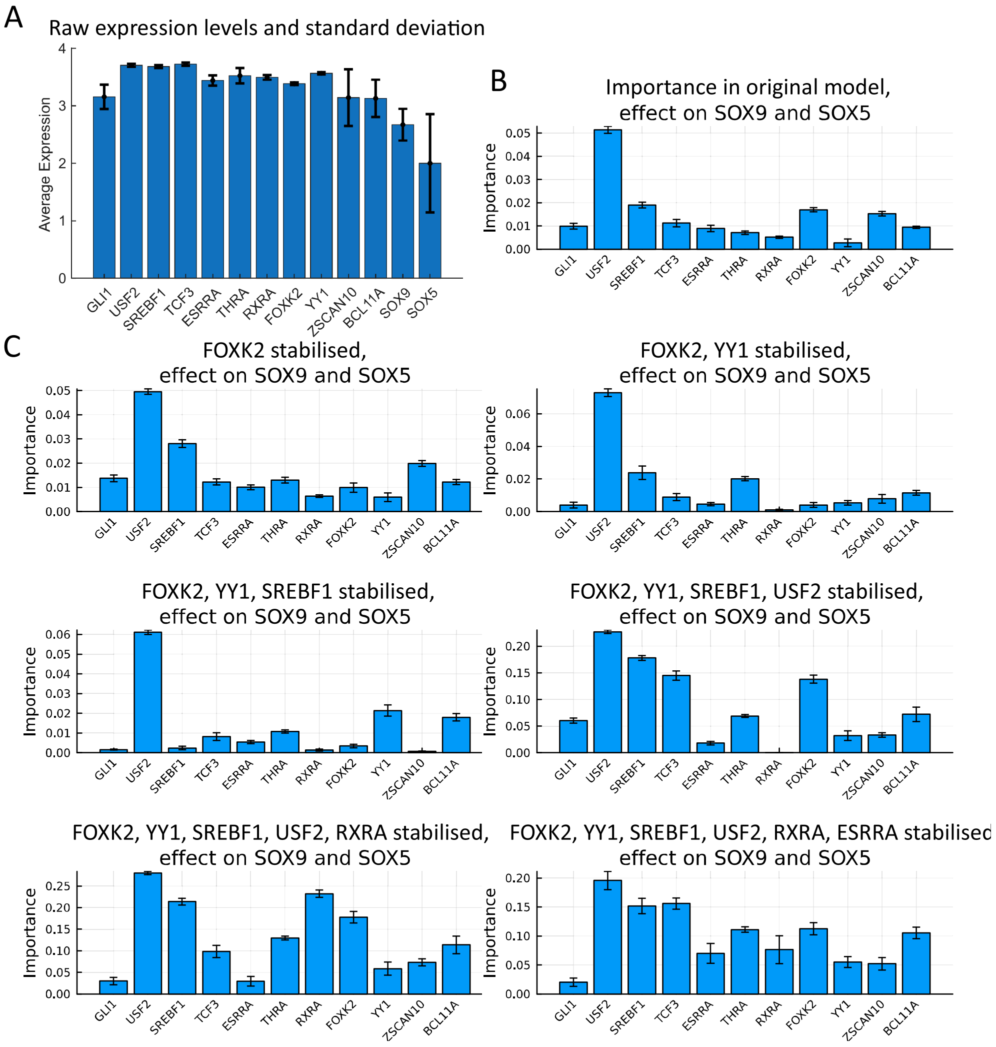


**Fig. C8**: Effects of stabilising gene expression on the importance score. **A**. Mean gene expression for all genes in the chondrogenesis networks with standard deviations, showing that some genes have very low variation in expression. **B**. Importance of transcription factors for driving SOX9 and SOX5 expression for the original model presented in Figure 3. **C**. Effects of iteratively stabilising the genes with lowest standard deviation in expression using Equation C1 on the importance metric computed using DANSE.

# Appendix D  Full list of selected transcription factors

## D.1  The chondrogenesis model

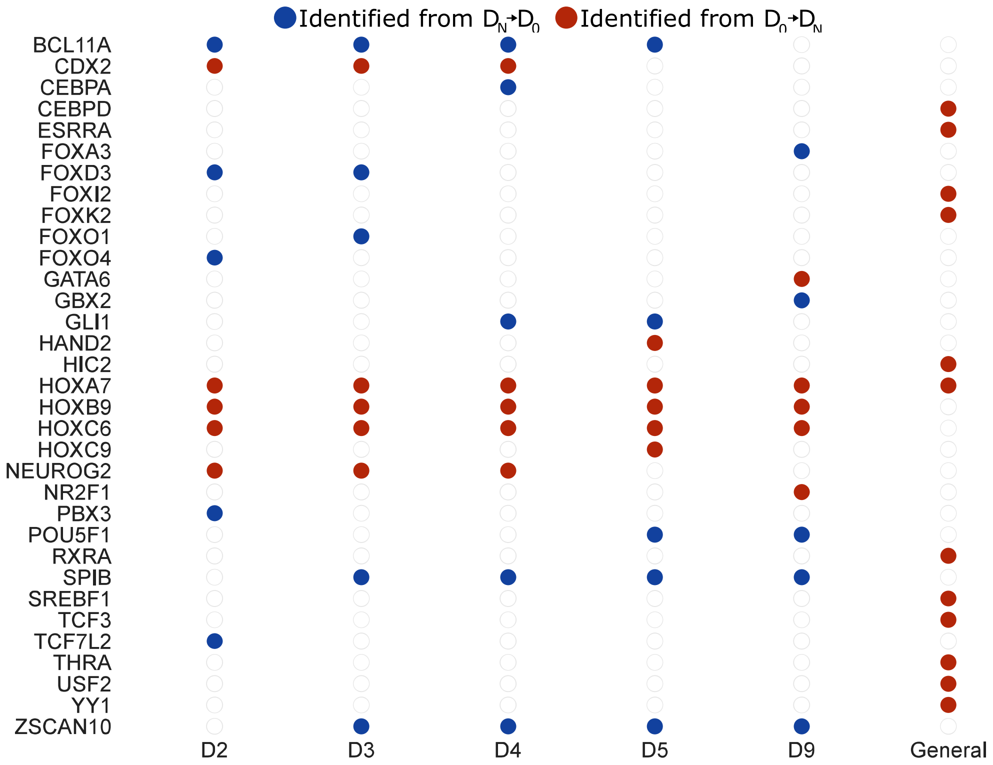


**Fig. D9**: Overview of selected genes for the model based on the data from [21].

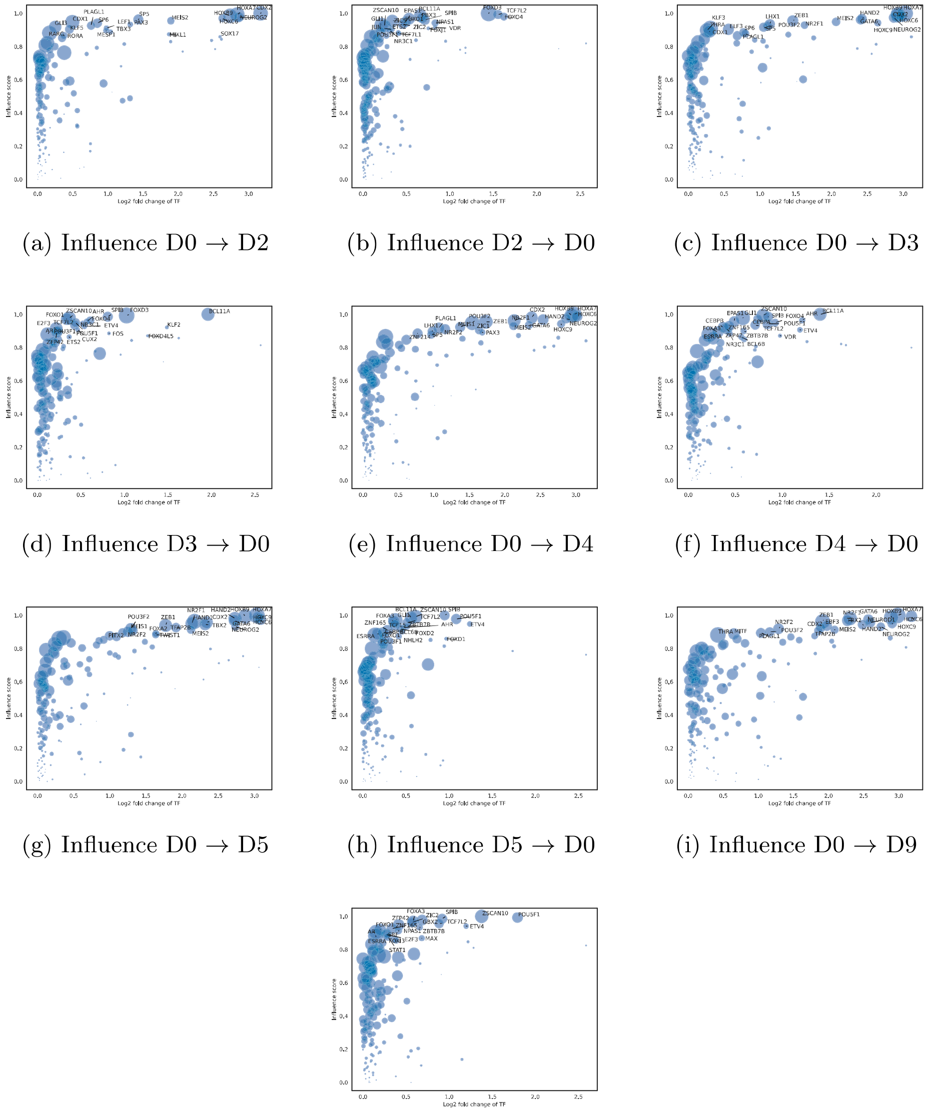

(a) Influence D0 → D2

(b) Influence D2 → D0

(c) Influence D0 → D3

(d) Influence D3 → D0

(e) Influence D0 → D4

(f) Influence D4 → D0

(g) Influence D0 → D5

(h) Influence D5 → D0

(i) Influence D0 → D9

(j) Influence D9 → D0

**Fig. D10**: *All influence plots for Kawata data. The horizontal axis indicates fold change of expression level, whereas the vertical axis indicates change of the influence score, as determined using ANANSE [11]. The size of each dot indicates the expression level in the target cell population.*

## D.2 The cardiogenesis model

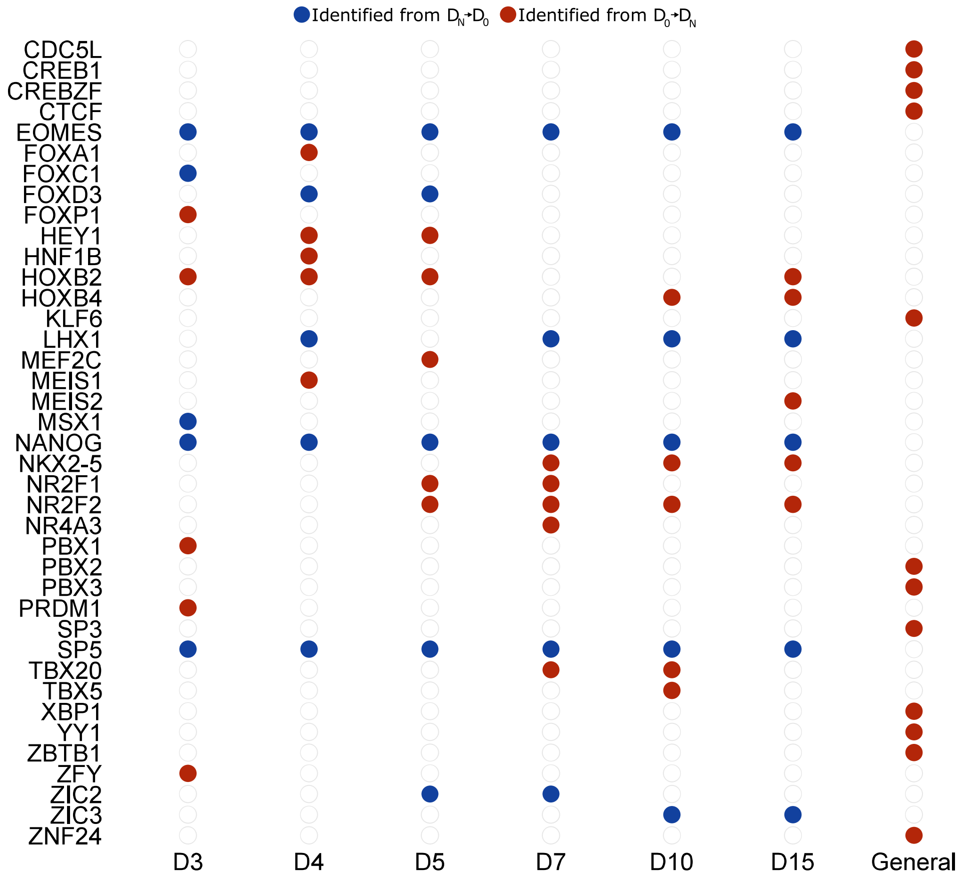


**Fig. D11**: Overview of selected genes for the model based on the data from [23].

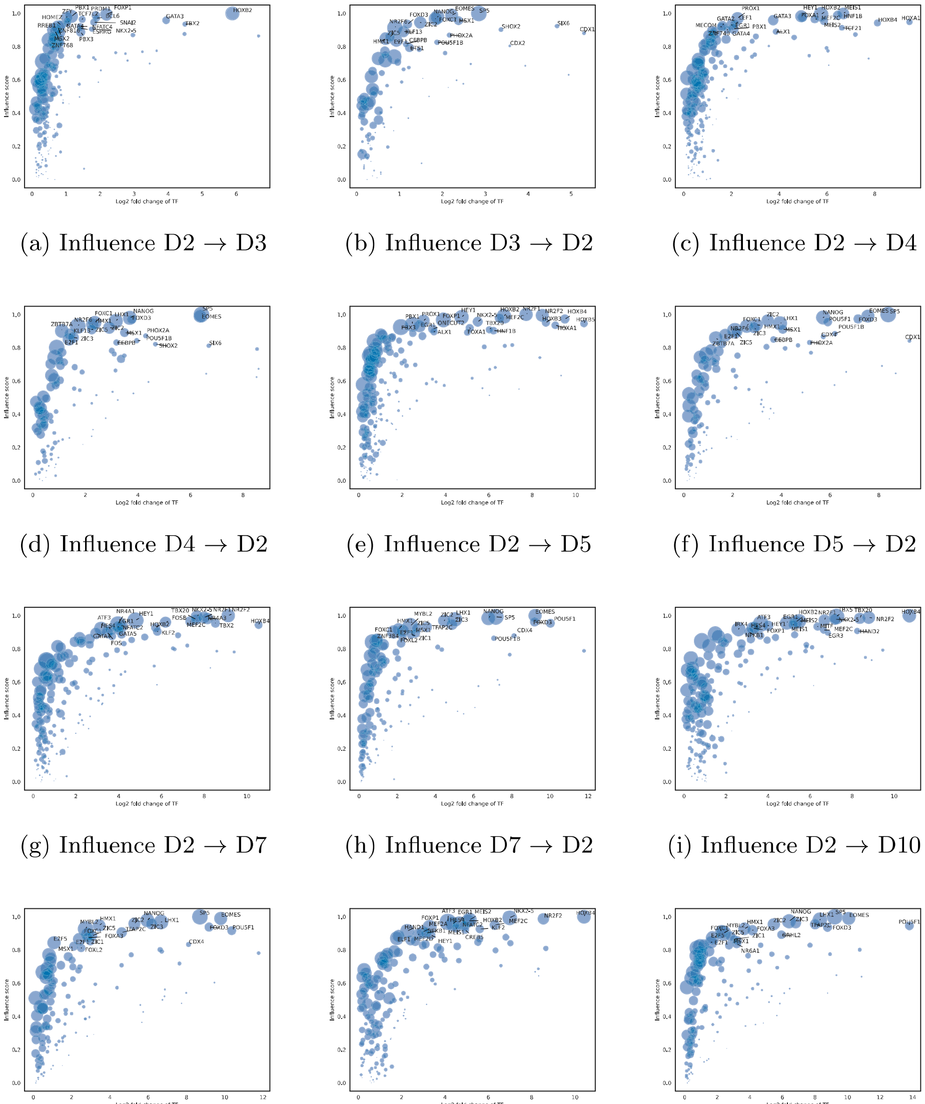

(a) Influence D2 → D3 (b) Influence D3 → D2 (c) Influence D2 → D4

(d) Influence D4 → D2 (e) Influence D2 → D5 (f) Influence D5 → D2

(g) Influence D2 → D7 (h) Influence D7 → D2 (i) Influence D2 → D10

(j) Influence D10 → D2 (k) Influence D2 → D15 (l) Influence D15 → D2

**Fig. D12**: *All influence plots for Meier data. The horizontal axis indicates fold change of expression level, whereas the vertical axis indicates change of the influence score, as determined using ANANSE [11]. The size of each dot indicates the expression level in the target cell population.*
